# From “player” to “viewer”: a survey on the willingness of video game players to alter their game consumption behavior

**DOI:** 10.3389/fpsyg.2026.1807531

**Published:** 2026-07-03

**Authors:** Xiao Gong, Jiangchao Wang, Yitong Cui, Zihao Wang, Buchao Wang

**Affiliations:** 1 Soochow University, Suzhou, China; 2 Harbin Institute of Technology, Harbin, China; 3 Zhejiang University of Finance and Economics Dongfang College, Haining, China; 4 Hebei Oriental University, Langfang, China; 5 Communication University of China Nanjing, Nanjing, China; 6 Lishui Technician College of Agriculture and Forestry, Lishui, China

**Keywords:** consumer behavior, esports live consumption, game consumption behavior, game consumption transformation, transtheoretical model (TTM)

## Abstract

**Background:**

As gameplay and esports live streaming increasingly coexist within the digital gaming ecosystem, it remains unclear whether players’ movement toward spectatorship represents behavioral substitution, complementary media use, or a broader restructuring of game-related consumption. Drawing on the Transtheoretical Model of behavior change, this study examines Chinese game users’ stage-based willingness to adjust their consumption from active gameplay toward esports live-streaming spectatorship.

**Methods:**

A cross-sectional survey was conducted among Chinese game consumers aged 18–45 years, yielding 675 valid responses. Participants were classified into five TTM stages: precontemplation, contemplation, preparation, action, and maintenance. The study used descriptive statistics, reliability analysis, CFA-based measurement validation, one-way ANOVA, and *post-hoc* tests to examine differences in gameplay habits, esports live-streaming consumption, consumption attitudes, behavioral willingness, and reasons for consumption adjustment across the five stages.

**Results:**

The stage distribution showed that 4.44% of participants were in precontemplation, 21.33% in contemplation, 29.33% in preparation, 25.78% in action, and 19.11% in maintenance. Descriptive results suggested a gradual shift from game-centered consumption to live-streaming-centered consumption from the early stages to the action stage, followed by partial stabilization or decline in the maintenance stage. ANOVA results showed significant stage differences in users’ attitudes toward playing games and watching esports live streams, current consumption habits, life-stage-related changes, support for esports live-streaming consumption, and opposition to esports live-streaming consumption. However, several game-related evaluations showed no significant stage differences, suggesting that basic gaming motivations and concerns may be shared across stages.

**Conclusion:**

The findings indicate that the player-to-viewer shift should not be understood as a simple or linear replacement of gameplay by live-streaming consumption. Instead, it is better conceptualized as a nonlinear, stage-dependent restructuring of game-related engagement. Users may reduce gameplay, combine playing and watching, or move between the two depending on life stage, available time, personal interests, and content quality. This study extends the TTM framework to digital gaming consumption and provides stage-specific implications for esports platforms, game companies, and live-streaming services.

## 1 Introduction

As a highly organized and intensely competitive professional sport utilizing video games as its medium, esports is experiencing unprecedented expansion in global influence and industry scale (Navarro-Lucena et al., 2025; Wollesen et al., 2025). In recent years, the landscape of global esports entertainment has undergone a structural transformation. Streaming ecosystems represented by platforms such as Twitch, YouTube Gaming, Douyu, and BiliBili in China have experienced rapid growth. The total number of global game live-streaming viewers has exceeded 1.5 billion, and the growth of market size and user viewing duration has begun to surpass that of traditional game hardware and software sales in many regions (Gamigion, 2025; Guo et al., 2024). The professionalization and league-based development of esports have further elevated gaming from a personal entertainment activity to a global sporting event simultaneously watched by millions of viewers (Navarro-Lucena et al., 2025; Molina et al., 2024). This thriving scene is reflected not only in the convergence of capital and audience engagement, but also in the fundamental evolution of user-game interaction (Majiri et al., 2025; Lee et al., 2025; Chalaby, 2024).

Currently, research on esports game consumption is shifting from a player-centric model to an audience-centric one, while academic research still needs to further catch up with industry practice. Early mainstream research on esports game consumption mainly analyzed players as active agents. Game consumption was understood as the economic resources, attention, and time that players invest in games (Jordan-Vallverdú et al., 2023; Pluss et al., 2019). Relevant studies have drawn on the Technology Acceptance Model (TAM) to explain how players form intentions to purchase and use games based on perceived usefulness and perceived ease of use (Bassiouni et al., 2019; Wang and Goh, 2017; Charness and Boot, 2016). Flow Theory has also been widely applied in game research to explain how players enter a state of deep immersion when challenge and skill are well balanced, thereby obtaining enjoyment, concentration, and intrinsic satisfaction from gameplay (Chen et al., 2025; Chen, 2024; Hou et al., 2019).

However, Flow Theory should not be understood as limited only to active gameplay. Recent research has extended the concept of flow to online game spectatorship and live-streaming contexts. For example, (Kim and Kim, 2022) showed that online game spectators may also experience flow while watching streamers’ broadcasts, and that streamer attributes, platform convenience, technological quality, perceived friendship, and streamer skills can influence spectators’ flow experience, psychological well-being, commitment, and loyalty. This indicates that esports spectatorship is not merely passive viewing, but can also involve concentration, emotional engagement, social connection, and psychological gratification.

As control is no longer the sole cornerstone of digital entertainment, the value logic of game consumption is expanding from direct operation to spectator-oriented engagement. Users may still remain connected to games even when they reduce direct gameplay, obtaining value through watching, learning, social interaction, emotional involvement, and identification with streamers, teams, or events. Existing research has already provided valuable insights into game spectatorship. For instance, (Orme, 2021) study of “just watchers” shows that individuals who do not regularly play video games may still actively engage with gaming culture through spectatorship. Their motivations include experiencing game narratives, reducing the “work” of gameplay, avoiding skill pressure or toxic gaming communities, and gaining vicarious enjoyment from watching others play. This suggests that spectatorship can function as a meaningful and culturally legitimate mode of game-related engagement rather than a secondary or marginal activity.

Therefore, this study adopts behavioral substitution as a theoretical framework to examine the psychological and behavioral transition from active gameplay to esports live-streaming spectatorship. This transition is not treated as a simple replacement of playing by watching, but as a stage-based process of consumption restructuring shaped by changing psychological needs, life conditions, and value perception. From an industry perspective, this shift also creates a clear business risk: if game companies and live-streaming platforms fail to accommodate users’ emerging spectator-like consumption needs, they may permanently lose players whose direct gameplay participation declines but whose interest in gaming culture remains. Accordingly, this study applies the Transtheoretical Model to explore the stage-based patterns through which users adjust their game-related consumption from “what I can do” toward “what I can see, learn, feel, and follow.”

## 2 Literature review

### 2.1 Conceptual explanation and research status of consumption behavior substitution

Behavioral substitution can be understood as a process of cognitive reassessment, psychological adaptation, and behavioral reallocation when individuals encounter changing internal needs or external constraints (Patey et al., 2022). Rather than referring only to the mechanical replacement of one activity by another, behavioral substitution involves the adjustment of satisfaction pathways through which individuals maintain psychological balance, emotional compensation, and value realization. When an existing behavior becomes costly, stressful, or less compatible with everyday life, individuals may redirect their engagement from a high-demand activity to an alternative form of participation with lower operational or cognitive requirements (Vansteenkiste et al., 2020). In the context of game consumption, this process may occur when users reduce direct gameplay and increasingly engage with esports live streaming as a spectator-oriented form of participation.

The possible movement from “player” to “spectator” is shaped by multiple psychological and contextual factors. Negative gaming experiences, including health-related risks and psychological strain (Zhou et al., 2025; Monteiro Pereira et al., 2022), social conflicts within gaming environments (Lin et al., 2023; Richard et al., 2023), and real-life resource constraints such as limited time, work commitments, and shifting interests (Vuorre et al., 2022; Mella et al., 2023; Liu et al., 2025), may weaken users’ willingness or ability to sustain intensive gameplay. At the same time, individuals may conduct a process of decisional balance by evaluating the perceived benefits and costs of continued gameplay and alternative forms of game-related consumption, such as esports live streaming (Zhang et al., 2024; Chen et al., 2024). Therefore, the transition from “playing” to “watching” should not be understood as a simple withdrawal from games, but as a possible reallocation of time, attention, emotional energy, and consumption resources within the broader gaming ecosystem.

This shift also involves changes in users’ value acquisition and identity positioning. In play-centered consumption, value is often derived from direct control, skill performance, achievement, and interactive participation. In audience-centered consumption, value may increasingly come from observation, social interaction, emotional companionship, admiration for skilled performers, and identification with streamers, teams, or fan communities. In this sense, the user’s value perception may shift from “what I can do” to “what I can experience, observe, and participate in through others.” The audience economy, supported by imagined closeness between viewers and hosts or contestants and by the indirect achievement obtained from watching others succeed, is reshaping the value chain of the gaming and esports industries (Kowert and Daniel, 2021; Andrew, 2019). Content creators and streamers increasingly function as important intermediaries between users and games, sometimes becoming central connectors in the relationship between gaming products, spectators, and communities (Thomason, 2025; Zhan et al., 2024; Nina, 2024).

Existing research has provided important insights into both gameplay and esports spectatorship. On the one hand, studies within the player-centered framework have examined gaming motivations, immersive experiences, and payment behaviors (Zhang et al., 2026; Linnemann et al., 2025; Usman et al., 2025; Holl et al., 2024; Kukshinov, 2024; Colombo et al., 2023; Zendle et al., 2023). On the other hand, audience-centered research has analyzed viewing motivations, donation behaviors toward streamers, and the formation of fan communities (Min et al., 2025; Pagáč et al., 2025; Guo et al., 2025; Tang et al., 2025; Jo and Shin, 2024; Xu and Ye, 2020). Recent gaming motivation research has further shown that players’ engagement is driven by multidimensional psychological needs, including challenge, autonomy, immersion, escapism, social connection, self-esteem, and consumption-related motivations such as virtual item purchase. These studies provide a useful foundation for explaining why users may reorganize their gaming engagement when direct gameplay no longer satisfies their psychological or practical needs (Duradoni et al., 2025; Gursesli et al., 2024; Martucci et al., 2023). However, these two research streams often treat players and spectators as relatively separate groups. Less attention has been paid to the dynamic mobility between these two roles, especially how existing players may reorganize their engagement from direct gameplay toward spectator-oriented consumption.

Accordingly, the player-to-spectator shift should not be framed as a purely linear or one-way process. Some users may substitute part of their gameplay with live-streaming consumption; others may combine gameplay and viewing in a complementary way; still others may restructure their engagement by becoming less involved in direct operation while remaining connected to gaming culture through spectatorship, social interaction, and symbolic consumption. This process may include identity repositioning from active operator to audience participant, as well as changes in psychological needs, behavioral patterns, and value preferences. In particular, when high operational barriers, social pressure, and fragmented personal time jointly affect users’ gaming experiences, the psychological mechanisms behind this role adjustment remain insufficiently explained.

In conclusion, the transition from player to spectator may involve behavioral substitution, complementary media use, and broader consumption restructuring. This perspective provides a stage-based theoretical foundation for examining how users evaluate gameplay and esports live streaming, how their behavioral transition intention is formed, and how this intention may or may not translate into actual live-streaming consumption across different stages of change.

### 2.2 Hinder and facilitate: multidimensional factors influencing behavioral substitution

The evolution from “player” to “audience” should be understood as a nonlinear and stage-dependent process shaped by multidimensional factors. The same factor may either hinder or facilitate consumption restructuring depending on users’ psychological readiness, social context, and resource conditions. Economic investment provides a typical example. On the one hand, sunk cost effects may create psychological and behavioral constraints that sustain continued gameplay (Hartig, 2017; Fujino et al., 2016; Westfall et al., 2012). On the other hand, excessive gaming-related spending may exhaust personal resources, trigger financial strain, and motivate users to reduce gameplay or seek lower-cost alternatives such as esports live streaming (Rockloff et al., 2020; Lohse, 2019; Wei et al., 2015; Gong S. et al., 2023; Gong M. et al., 2023; Petrovskaya and Zendle, 2022; Linnemann et al., 2025; Kolandai-Matchett and Wenden Abbott, 2021). Therefore, the factors influencing behavioral substitution do not operate in a fixed direction. Instead, they may function as either barriers or drivers at different stages of behavioral change.

At the individual psychological and physiological level, excessive immersion, problematic gaming, and gaming-related dependence may create strong behavioral inertia, making it difficult for users to reduce gameplay even when they recognize its negative consequences (Xu et al., 2024; Wu et al., 2022; Antons et al., 2023; Griffiths, 2012). Strong identification with one’s gaming skills may also reduce willingness to shift toward spectator-oriented consumption, because the player’s self-worth is partly anchored in active performance and direct control (Dai et al., 2024; Wang et al., 2021). However, health concerns such as prolonged sitting, visual fatigue, and competitive pressure may also motivate users to reconsider the sustainability of intensive gameplay (Palanichamy et al., 2020; Prochaska et al., 1997). Higher education and stronger self-management capacity may further support users’ reflective evaluation of their consumption habits and alternative forms of engagement (Karlen et al., 2021; Demetriou et al., 2020; Guerra-Carrillo et al., 2017). In addition, physiological changes associated with age, such as slower reaction speed or reduced tolerance for high-intensity action games, may encourage some users to maintain their connection to gaming culture through less operationally demanding forms of participation.

At the product and content experience level, high-quality game design, including challenging mechanics, rich narratives, and strong social systems, can strengthen immersion, community belonging, and user retention (Ascarza et al., 2025; Lagrange, 2025; Games, 2020). In-game purchases may also provide direct virtual assets, skill advantages, or visible status symbols, whereas live-streaming rewards are often more emotional, symbolic, and relationship-oriented (Ungureanu and Schauerte, 2025; Ruck, 2020; Fields and Cotton, 2011; Luo et al., 2026; Bai et al., 2025; Zhang et al., 2023). This difference in perceived achievement may hinder the transfer from gameplay consumption to live-streaming consumption (Ungureanu and Schauerte, 2025; An et al., 2024; Cai et al., 2022). Nevertheless, when gameplay produces diminishing marginal utility because of repetition, frustration, or declining interest, esports live streaming may become more attractive by offering entertainment value, learning opportunities, low-threshold viewing, and streamer charisma (Qiao et al., 2025; Huang et al., 2024; Jiang et al., 2024; Jia et al., 2024; Zhang et al., 2024). The professionalism, uncertainty, and drama of esports competitions may also make esports viewing comparable to traditional sports spectatorship and attract both core players and peripheral audiences (Pedraza-Ramirez et al., 2025; Wollesen et al., 2025).

At the social-environmental interaction level, strong social ties within games may function as important exit barriers. Players may delay or resist reducing gameplay because leaving the game could weaken online friendships, team identity, or status within the gaming community (Alexandrovsky et al., 2024). However, esports live-streaming platforms also provide alternative social spaces. Bullet-screen comments, fan communities, information sharing, and streamer-centered interaction can offer companionship, social presence, and group identity (Zou and Mu, 2024; Soni and Sarkar, 2023; Li et al., 2021). For users who wish to maintain a connection to gaming culture but cannot sustain high levels of operational involvement, watching live streams may provide a lightweight way to remain socially and emotionally connected to the gaming community (Li et al., 2020).

At the economic and resource constraint level, disposable time, financial capacity, and everyday life structure shape users’ ability to maintain either gameplay or live-streaming consumption. Limited time can restrict both intensive gaming and prolonged live-streaming viewing, while direct costs such as tipping and subscriptions and indirect costs such as internet data charges may impede continued engagement with live-streaming platforms (Li et al., 2020; Kende, 2021). Users with greater economic resources may be more likely to experiment with and sustain live-streaming consumption (Zhang et al., 2024). At the same time, the fragmentation of leisure time may make esports live streaming easier to integrate into everyday routines than long gaming sessions, thereby facilitating a partial shift from direct gameplay to viewing-oriented participation (Li et al., 2024a; Li et al., 2024b; Li et al., 2024c).

Taken together, these multidimensional factors generate both push and pull forces in the reorganization of game-related consumption. Game negative pressure, health concerns, social conflict, declining interest, and real-life constraints may push users away from intensive gameplay. In contrast, the entertainment value, learning function, social interaction, and emotional companionship of live streaming may pull users toward spectator-oriented engagement. However, this process is not uniform across all users. Depending on users’ stage of readiness, the same factor may operate as a barrier, a facilitator, or both. This stage-dependent logic provides a theoretical basis for examining how behavioral transition intention emerges and why such intention may translate into esports live-streaming consumption for some users but not for others.

### 2.3 Theoretical framework for behavior change

**Figure 1:**
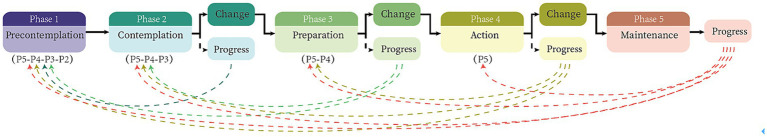
TTM theoretical model diagram.

This study employs the transtheoretical Model (TTM) as an integrative analytical framework to explore the behavioral transition process from “operator” to “spectator” driven by multidimensional factors. TTM categorizes individuals into distinct groups based on their evolving states during behavioral change, thereby conceptualizing behavioral transformation (Prochaska et al., 1997). The model posits that positive behavioral substitution occurs through five distinct phases: (1) No intention to change, (2) Intention to change, (3) Preparation phase, (4) Action phase, and (5) Maintenance phase (Rouhani-Tonekaboni et al., 2024). This study utilizes an adapted TTM to examine consumption patterns across different consumer stages (Nakabayashi et al., 2020). TTM has been empirically validated as a model for behavioral change mechanisms (Figure 1). In reality, consumer behavior is influenced by multiple factors and barriers, resulting in nonlinear behavioral transitions. For instance, within TTM, consumers may regress to earlier stages (e.g., users who previously preferred in-game purchases may revert to in-game spending) or remain at a stage for extended periods due to barriers (e.g., lack of financial resources or time for sustained behavior), before progressing to subsequent phases. Overall, TTM’s stage-based classification facilitates identification of critical psychological nodes in behavioral transition processes, while its acknowledgment of nonlinear transitions supports potential regression phenomena during the “player” to “spectator” shift.

By integrating the conceptual framework of behavioral substitution with the phased and measurable nature of TTM, this study develops a dynamic analytical model. The model not only delineates specific behavioral differences across substitution stages, but also systematically identifies key factors that influence decision-making balance and self-efficacy at each stage, demonstrating their role as either barriers or facilitators, and how individuals formulate adaptive strategies accordingly.

### 2.4 Research questions

Based on the theoretical framework, this study aims to explore the following questions:

RQ1: What factors shape the shift in value perception from “operational control” to “spectator-oriented engagement” across TTM stages?

RQ2: How do individual, product-related, social, and resource-related factors hinder or facilitate this consumption shift?

RQ3: How do consumption patterns, including type, frequency, amount, and time allocation, differ across TTM stages?

RQ4: What stage-specific factors should be considered when developing adaptive engagement and retention strategies?

## 3 Methods

### 3.1 Target audience

The study group comprises electronic game users aged between 18 and 45 within China. The selection of this population group is primarily based on the following considerations (Bao et al., 2025): Firstly, individuals within this age bracket generally possess independent economic control and a high level of digital media literacy, the core audience for gaming and live streaming consumption (Li et al., 2024a; Li et al., 2024b; Li et al., 2024c; Li et al., 2023); Secondly, they are in a life stage with greater personal freedom, where their consumption decisions are less influenced by external factors, thus more accurately reflecting individual preferences (Atkinson, 2012). Data from 2020 shows that the number of China’s online video users has reached 944 million, accounting for 95.4% of the total number of Internet users. This indicates that this group has an extremely high exposure rate and acceptance of online video content, providing an ideal empirical context for this study.

### 3.2 Questionnaires and scales

This study employed a cross-sectional questionnaire survey. The questionnaire was developed using established measurement scales and systematic literature reviews, aiming to assess key constructs associated with the substitution process of “game-streaming” behavior. Each item in the questionnaire was rated on a 5-point Likert scale (1 = strongly disagree, 5 = strongly agree) to enhance discriminative ability and minimize response bias from neutral options. The questions covered the following research topics:

1. The stages of the Theory of Change Model (TTM) are defined as follows: Pre-change (no intention to change), Intention to Change (intending to change within 6 months), Preparation to Change (planning to change within 30 days), Action to Change (already changed but less than 6 months), and Maintenance to Change (already changed for over 6 months). Participants are asked to select the stage that best describes their current state, and this classification is applied throughout the entire questionnaire analysis.
2. Sociodemographic information, including gender, age, weight, education, current status, and nationality, to describe the sociodemographic background (see Table 1).
3. The proportion of time spent on gaming and live streaming, revealing the relative time allocation between ‘gaming’ and ‘live streaming’ among participants at different stages (see Table 2).
4. Consumer spending preferences. It examines the allocation ratio of money and time between in-game purchases and live-streaming consumption across different stages, as well as the duration of individual play sessions or viewing (see Table 3).
5. Frequency of consumption, recording the number of times participants spend in games and live streams (see Table 4)
6. The study examines consumption fluctuations during life stage transitions, analyzing shifts in gaming and live-streaming spending proportions among participants entering new life phases (e.g., academic advancement or employment) (see Table 5).
7. The reasons for supporting gaming include Ambition (Holl et al., 2024), Hobby (Richard et al., 2025), Personalization (Alhasani et al., 2026), Protection (Hu et al., 2024), Accomplishment (Kiralj Lacković, 2024), Vanity (Koles et al., 2025), Avoid Duplication (Gursesli et al., 2024), New Gameplay (Ristianto et al., 2025), Purchase duration (Jiao et al., 2022), Purchase efficiency (Li et al., 2025), Investment and financial management (Christoph et al., 2023), Same group (Gursesli et al., 2024), Belonging (Ballou et al., 2024) (see Table 6).
8. Reasons for opposing gaming include Challenge (Mills et al., 2023), Creativity (Cheng, 2025), Operability (Magnusson et al., 2024), Sociality (Gonçalves et al., 2023), Free (Zhang et al., 2025), Rewarding (Zhou et al., 2025), Ethical issues (Laperdrix et al., 2022), Gaining weight (Simons et al., 2015), Poor lung health (Mishra et al., 2025), Joint pressure (Alqabbani, 2025), Poor sleep (Liu et al., 2025), Declining vision (Wang et al., 2025), Emotional instability (Lin et al., 2020), Poor social relationships (Slack et al., 2025), Lack of prospects in life (Fraser et al., 2023), Conformity (Chung et al., 2025), Forbiddingness (Kolandai-Matchett et al., 2021) (see Table 7).
9. The reasons for participants’ support of watching live broadcasts included: The physical skills of athletes (Rietz and Hallmann, 2022), Acquisition of knowledge (Wang, 2023), the psychology of conformity (Wooyoung et al., 2024), Dramatic (Ma et al., 2021), Physical health and Mental health (Rietz and Hallmann, 2022), Convenience (Jiang et al., 2024) (see Table 8).
10. Reasons for opposing watching live streaming: Content format, Spiritual emotion, Moral issues, Convenience (Jiang et al., 2024) (see Table 9)

**Table 1:** Demographic characteristics.

Variable	Category	Precontemplation	Contemplation	Preparation	Action	Maintenance
Gender	Male	40.0%	39.5%	**48.4%**	41.3%	*32.5%*
	Female	60.0%	60.4%	*51.5%*	58.6%	**67.4%**
Age	18–25	**100.0%**	77.0%	54.5%	46.5%	*41.8%*
	26–30	*0.0%*	20.8%	42.4%	48.2%	**51.1%**
	31–35	*0.0%*	*0.0%*	3.0%	**3.4%**	2.3%
	>35	*0.0%*	2.0%	*0.0%*	1.7%	**4.6%**
Nationality	Chinese nationality	*90.0%*	93.7%	**96.9%**	94.8%	90.6%
	Other	**10.0%**	6.2%	*3.0%*	5.1%	9.3%
Education level	High School/Secondary Vocational	**10.0%**	*0.0%*	1.5%	1.7%	2.3%
	Junior college	**50.0%**	29.1%	9.0%	*8.6%*	11.6%
	Undergraduate	*30.0%*	64.5%	77.2%	81.0%	**81.3%**
	Master	**10.0%**	6.2%	*4.5%*	5.1%	4.6%
	Doctor	*0.0%*	*0.0%*	**7.5%**	3.4%	*0.0%*
Working conditions	Junior college	**50.0%**	27.0%	7.5%	*6.8%*	11.6%
	Undergraduate	*40.0%*	45.8%	45.4%	44.8%	**51.1%**
	Master	**10.0%**	6.2%	6.0%	*1.7%*	4.6%
	Doctor	*0.0%*	**2.0%**	1.5%	1.7%	*0.0%*
	Work (1–3 years)	*0.0%*	6.2%	**24.2%**	20.6%	16.2%
	Work (3–5 years)	*0.0%*	4.1%	9.0%	**18.9%**	11.6%
	Work (>5 years)	*0.0%*	**6.2%**	6.0%	5.1%	4.6%
	High School/Secondary vocational	*0.0%*	**2.0%**	*0.0%*	*0.0%*	*0.0%*
Education type	Academic	40.0%	*31.2%*	**51.5%**	48.2%	51.1%
	Technique	60.0%	**68.7%**	*48.4%*	51.7%	48.8%
Residence	Urban	*80.0%*	**87.5%**	83.3%	82.7%	86.0%
	Countryside	**20.0%**	*12.5%*	16.6%	17.2%	13.9%

Bold = highest share, italic = The lowest share.

**Table 2:** Subjects’ attitudes toward playing games and watching esports game live broadcasts.

Variable	Precontemplation	Contemplation	Preparation	Action	Maintenance
Always played games, did not watch live broadcasts	**72.30%**	40.80%	14.50%	6.70%	*0.00%*
Frequently played games, rarely watched live broadcasts	22.90%	**45.90%**	13.80%	4.50%	*0.00%*
Occasionally played games, rarely watched live broadcasts	4.80%	8.50%	18.90%	**63.90%**	*0.00%*
Rarely played games, occasionally watched live broadcasts	*0.00%*	4.80%	**51.30%**	16.90%	6.80%
Rarely played games, frequently watched live broadcasts	*0.00%*	*0.00%*	1.50%	6.90%	**18.40%**
Didn’t play games, Always watched live broadcasts	*0.00%*	*0.00%*	*0.00%*	1.10%	**74.80%**

Question: Did you currently play more games or watch esports live broadcast more? Bold = highest share, italic = The lowest share.

**Table 3:** Current habits of the subjects.

Variable	Category	Precontemplation	Contemplation	Preparation	Action	Maintenance
More money spent on playing games/ watching live broadcasts	Playing games	40.00%	**72.90%**	45.40%	*5.10%*	6.90%
	Watching live broadcasts	*0.00%*	14.50%	36.30%	**75.80%**	67.40%
	Equal	**20.00%**	10.40%	15.10%	13.70%	*6.90%*
	None	**40.00%**	*2.00%*	3.00%	5.10%	18.60%
More times spent on playing games/ watching live broadcasts	Playing games	70.00%	**77.00%**	57.50%	*8.60%*	13.90%
	Watching live broadcasts	*0.00%*	16.60%	34.80%	**81.00%**	72.00%
	Equal	**30.00%**	*6.20%*	7.50%	10.30%	13.90%
Single duration of playing games	<30 min	10.00%	*6.20%*	15.10%	25.80%	**46.50%**
	30 min-60 min	20.00%	22.90%	*10.60%*	**53.40%**	25.50%
	1 h-3 h	50.00%	**52.00%**	33.30%	*8.60%*	23.20%
	3 h-5 h	10.00%	14.50%	**31.80%**	10.30%	*4.60%*
	5 h-8 h	**10.00%**	4.10%	4.50%	1.70%	*0.00%*
	>8 h	*0.00%*	*0.00%*	**4.50%**	*0.00%*	*0.00%*
Single duration of watching live broadcasts	<30 min	**100.00%**	45.80%	16.60%	*5.10%*	23.20%
	30 min-60 min	*0.00%*	**37.50%**	27.20%	12.00%	23.20%
	1 h-3 h	*0.00%*	8.30%	24.20%	**39.60%**	30.20%
	3 h-5 h	*0.00%*	8.30%	**24.20%**	22.40%	11.60%
	5 h-8 h	*0.00%*	*0.00%*	3.00%	**12.00%**	6.90%
	>8 h	*0.00%*	*0.00%*	4.50%	**8.60%**	4.60%

Bold = highest share, italic = lowest share.

**Table 4:** Subjects’ consumption status and willingness.

Variable	**Precontemplation**	**Contemplation**	**Preparation**	**Action**	**Maintenance**
Frequency of consumption	x	3.20	3.09	3.35	3.03
The frequency of farming gifts during live game events	x	3.19	2.97	4.07	3.53
The consumption frequency of peripheral cultural and creative products issued during gaming events	x	3.27	3.30	3.10	2.95
The consumption frequency of game event tickets	x	2.94	2.70	2.72	2.70
The consumption frequency of game event battle orders	x	3.48	3.59	3.28	3.07
The consumption frequency of game event win-loss betting	x	3.13	2.89	3.62	2.91
Willingness to consume	2.76	2.95	3.22	3.13	2.97
The willingness to consume peripheral cultural and creative products in game events	3.30	3.21	3.42	3.24	2.70
The willingness to farm gifts in game events	2.40	2.50	2.82	3.09	3.33
The willingness to consume tickets in game events	2.20	2.81	3.08	2.98	2.63
The willingness to consume battle orders in game events	3.20	3.17	3.68	3.45	3.14
The willingness to consume win-loss betting in game events	2.70	3.04	3.12	2.90	3.05

The darker the color, the larger the value; the lighter the color, the smaller the value.

**Table 5:** The changes of the subjects in the new stage of their lives.

Variable	Precontemplation	Contemplation	Preparation	Action	Maintenance
Gaming time reduced by more than half (50–99%)	*10.00%*	37.50%	34.80%	**48.20%**	30.20%
Gaming time reduced by less than half (less than 50)	*30.00%*	35.40%	**36.30%**	34.40%	34.80%
No change in gaming time	**40.00%**	*14.50%*	18.10%	15.50%	27.90%
Increased gaming time	**20.00%**	12.50%	10.60%	*1.70%*	6.90%

**Table 6:** Knowledge, attitudes and beliefs about supporting consumption in playing games.

Variable	Category	**Precontemplation**	**Contemplation**	**Preparation**	**Action**	**Maintenance**
Support for continuing to play games and consumption		3.12	3.23	3.47	3.22	3.22
Psychological needs		3.43	3.49	3.65	3.37	3.22
I want to be the best in this game.	Ambition	3.50	3.46	3.62	3.45	3.35
I want to spend money on my gaming hobby.	Hobby	3.50	3.58	3.70	3.40	3.30
I want to personalize my character and the things I have built.	Personalization	3.60	3.75	3.74	3.50	3.16
I want to protect what I have already obtained in the game and not lose it again	Protection	3.50	3.50	3.68	3.43	3.09
I want to complete a level/task/building, etc.	Accomplishment	3.60	3.35	3.77	3.36	3.37
I want to showcase my achievements in the game and flaunt my accomplishments to everyone.	Vanity	2.90	3.27	3.41	3.10	3.05
The game		3.24	3.32	3.59	3.28	3.38
Spending money can help me avoid repeatedly completing a certain game task.	Avoid duplication	3.20	3.29	3.62	3.24	3.40
Spending money can assist me in unlocking new gameplay or game content.	New gameplay	3.50	3.42	3.68	3.41	3.44
I wish to continue playing this game.	Purchase duration	3.20	3.35	3.52	3.33	3.26
Spending money can accelerate my gaming experience.	Purchase efficiency	3.30	3.23	3.58	3.31	3.42
Spending money is an investment; the items I purchase will become increasingly valuable.	Investment and financial management	3.00	3.29	3.56	3.12	3.37
Social needs		2.70	2.90	3.16	3.01	3.06
Spending in games is to better maintain interpersonal relationships.	Same group	2.70	2.96	3.26	3.14	3.17
Spending in games is because I am in a romantic relationship or have deep feelings for the guild.	Belonging	2.70	2.83	3.06	2.88	3.05

The darker the color, the larger the value; the lighter the color, the smaller the value.

**Table 7:** Knowledge, attitudes and beliefs about opposing consumption in playing games.

Variable	Category	Precontemplation	Contemplation	Preparation	Action	Maintenance
Oppose continued gaming and consumption		3.25	3.28	3.30	3.34	3.36
The game		2.93	3.39	3.44	3.19	3.29
The game is completely unchallenging, which gives me no motivation to spend.	Challenge	2.80	3.38	3.26	3.00	3.02
The game is entirely lacking in innovation, which gives me no motivation to spend.	Creativity	2.80	3.48	3.58	3.16	3.35
The game has no operability, which gives me no motivation to spend.	Operability	2.90	3.35	3.48	3.09	3.35
The game lacks social gameplay, making me feel lonely and bored, thus I do not wish to spend.	Sociality	2.70	3.35	3.38	3.17	3.30
Playing games requires payment, so I do not play.	Free	2.70	3.29	3.41	3.10	3.19
Playing games does not earn money, so I do not play.	Rewarding	3.00	3.17	3.03	3.12	3.00
Some games are too offensive, so I do not play.	Ethical issues	3.60	3.73	3.95	3.69	3.79
Physiological health		3.48	3.46	3.53	3.54	3.52
Playing games for too long causes me to eat or drink more unhealthily foods, which in turn can cause me to gain weight.	Gaining weight	3.20	3.25	3.33	3.29	3.23
Playing games for too long leads to more cigarette smoke, which is not good for the lungs.	Poor lung health	3.40	3.25	3.45	3.22	3.30
Playing games for too long leads to prolonged sitting, which is not good for the joints.	Joint pressure	3.60	3.60	3.76	3.78	3.60
Poor sleep due to playing games for too long	Poor sleep	3.50	3.56	3.45	3.55	3.65
Playing games for too long leads to vision loss	Declining vision	3.70	3.65	3.65	3.86	3.81
Mental health		3.30	3.22	3.16	3.41	3.36
Playing games can make me irritable, depressed, and emotionally unstable.	Emotional instability	3.40	3.40	3.32	3.55	3.37
Playing games makes me feel lonely and less connected with my family, friends, and people around me.	Poor social relationships	3.30	3.19	3.12	3.38	3.30
Playing games make me feel hopeless in life and not want to work or study.	Lack of prospects in life	3.20	3.06	3.03	3.29	3.40
Interpersonal society		3.30	3.03	3.08	3.21	3.28
My friends, relatives, and loved ones do not play this game, so I will not play it either.	Conformity	3.50	3.13	3.03	3.26	3.37
The social group I belong to does not permit me to play games	Forbiddenness	3.10	2.94	3.12	3.16	3.19

The darker the color, the larger the value; the lighter the color, the smaller the value.

**Table 8:** Supported watching esports live broadcast and consumption.

Variable	Precontemplation	Contemplation	Preparation	Action	Maintenance
Support live broadcast consumption	3.41	3.58	3.92	3.99	3.78
The physical skills of athletes	3.57	3.85	4.09	4.17	3.81
The host’s superb operational level is what I admire.	3.50	3.75	4.03	4.10	3.72
The host’s proficient operational level is what I admire.	3.60	3.83	4.08	4.17	3.84
The host’s creative methods are what I like.	3.60	3.96	4.15	4.22	3.88
Entertainment and escapism	3.40	3.58	3.64	3.71	3.47
Passing the time	3.40	3.58	3.64	3.71	3.47
Acquisition of knowledge	3.67	3.88	4.14	4.16	3.85
Watching esports live broadcasts can deepen my understanding of game strategies and operations	3.60	3.79	4.08	4.10	3.84
By watching esports live broadcasts, I can learn about game operation techniques	3.70	3.90	4.21	4.14	3.84
By watching esports live broadcasts, I can learn about the explanation of basic game knowledge	3.70	3.94	4.12	4.24	3.88
The psychology of conformity	3.30	3.38	3.95	3.84	3.72
Those who play games with me all watch game live streams.	3.30	3.42	4.00	3.95	3.74
Many of my close companions are watching game live streams.	3.30	3.33	3.91	3.72	3.70
Dramatic	3.57	3.83	4.21	4.09	4.02
I enjoy the drama of intense esports competitions.	3.50	3.65	4.11	3.90	3.95
I appreciate the uncertainty brought by evenly matched esports competitions.	3.60	3.83	4.23	4.14	4.07
I like that in esports competitions, nothing is known about the outcome until the very end.	3.60	4.00	4.29	4.24	4.02
Physical health	3.10	3.29	3.69	3.88	3.68
I feel more energetic.	3.10	3.48	3.71	3.93	3.79
My weight has improved.	3.00	3.29	3.68	3.90	3.74
My joints are more flexible, and my immunity has strengthened.	3.20	3.17	3.65	3.86	3.58
My daily routine has become more regular.	3.10	3.21	3.70	3.83	3.61
Mental health	3.33	3.35	3.70	3.90	3.72
My emotions have become more stable.	3.40	3.38	3.70	3.86	3.74
I feel more love.	3.40	3.40	3.73	3.93	3.70
I am filled with hope and motivation for life.	3.20	3.29	3.67	3.90	3.72
Convenience	3.35	3.48	3.70	3.88	3.65
Live broadcasts can be viewed at any time	3.70	3.83	4.06	4.16	3.95
Live broadcasts can be viewed from anywhere	3.70	3.77	4.11	4.17	3.93
There are many types of live broadcasts, making it easy for me to find what I want to watch	3.50	3.71	4.09	4.19	3.88

The darker the color, the larger the value; the lighter the color, the smaller the value.

**Table 9:** Objected to watching esports live broadcast and consumption.

Variable	**Precontemplation**	**Contemplation**	**Preparation**	**Action**	**Maintenance**
Oppose live broadcast consumption	2.51	2.61	2.55	3.00	2.84
Content format	3.56	3.33	3.42	2.94	3.09
There is no sense of operation at all.	3.60	3.46	3.41	3.03	3.19
There is no feeling of playing together.	3.60	3.50	3.50	2.93	3.14
These players or streamers are not as good as I am, and I disdain to watch.	3.70	3.19	3.42	2.95	3.05
I do not like the commentary by the streamers.	3.50	3.21	3.32	2.97	3.07
I do not like the artistic style of the live streaming room.	3.40	3.29	3.44	2.83	3.02
Spiritual emotion	3.40	3.25	3.26	2.89	3.09
It makes me feel like I do not have a sense of gain.	3.20	3.31	3.33	2.97	3.14
It made me feel like I was completely wasting my time.	3.60	3.21	3.14	2.81	3.00
Makes me emotionally unstable.	3.40	3.23	3.32	2.90	3.12
Moral issues	3.50	3.67	3.79	3.43	3.47
The comments in the live broadcast make me feel uncomfortable.	3.60	3.67	3.70	3.28	3.42
The unscrupulous advertisements in the live broadcast make me feel	3.50	3.75	3.91	3.53	3.58
The gambling activities in the live broadcast make me feel uncomfortable.	3.40	3.60	3.77	3.47	3.40
Convenience	3.48	3.38	3.39	2.85	3.06
The data consumption while watching live broadcasts is too high.	3.50	3.40	3.41	2.91	3.07
Watching live broadcasts require a long period of stable signal.	3.50	3.35	3.41	2.85	3.00
Due to limitations of the device, I do not enjoy watching live broadcasts.	3.50	3.38	3.38	2.78	3.12
The live broadcasts of competitions are at specific times, and I can never manage to catch them, so I do not enjoy watching.	3.40	3.38	3.36	2.85	3.05

The darker the color, the larger the value; the lighter the color, the smaller the value.

The research team used a mature online survey software “Wenjuanxing” to collect the basic data for this research. Since Chinese people do not often use email, the team distributed questionnaires through Chinese social networking platforms such as Bilibili, Baidu Tieba, and Xiaohongshu to improve response rates. The survey lasted for 12 weeks from September to December 2024, and a total of 956 responses were received. The average time spent on the questionnaire was 12 min and 36 s, which is relatively long for an online survey.

### 3.3 Translation and pre-test procedures

To ensure the conceptual accuracy of the English source scale in the Chinese cultural context, all adapted scales adhered to a rigorous translation-back translation procedure. Initially, two bilingual researchers independently translated the original English version into Chinese. Subsequently, a third translator, who was not previously exposed to the source scale, performed a back-translation of the Chinese version into English. The research team compared the back-translated version with the original version, discussed and revised entries with ambiguities until consensus was reached. The revised full questionnaire underwent a small-scale (n = 30) pre-test prior to formal distribution to assess item clarity, comprehension difficulty, and completion time, with minor refinements to certain wording based on feedback.

### 3.4 Semantic content analysis

The research team posed an open-ended question: “What factors might lead you to reduce the time and frequency of watching esports live streams in the future?” Participants were encouraged to provide open-ended responses. A content analysis method (Eun et al., 2024) was employed, which involved the following steps: coding the textual data sentence by sentence, inducing initial concepts, merging similar concepts to form higher - level themes, and finally interpreting the frequency and implications of different themes to supplement quantitative analysis and gain deeper insights into the underlying considerations of user decisions.

For the quantitative analysis, this study further tested whether the observed mean differences across the five TTM stages were statistically significant. One-way ANOVA was conducted with TTM stage (1–5) as the grouping variable and the main measurement dimensions corresponding to Tables 1–10 as dependent variables. Levene’s test was first used to examine the homogeneity of variance. When the homogeneity assumption was met, Tukey HSD post-hoc tests were applied; when the assumption was violated, Games-Howell post-hoc tests were used. Partial eta squared (np2) was reported as an effect size, with values around 0.01, 0.06, and 0.14 interpreted as small, medium, and large effects, respectively. This procedure was added to avoid relying solely on descriptive means and to identify which stage-based differences were statistically supported.

**Table 10:** Subjects’ preferences for game products and esports live streaming.

Variable	Precontemplation	Contemplation	Preparation	Action	Maintenance
I will spend more money due to the brand recognition of the game or the players.	3.40	3.38	2.94	3.07	3.86
The stronger the gaming experience, the more money I will spend.	3.50	3.54	3.59	3.22	3.54
The stronger the social interaction within the game, the more money I will spend.	3.29	3.57	3.44	3.21	3.30
The smoother the software interaction of the game, the more money I will spend.	3.20	3.40	2.46	3.24	3.54
The stronger the sensory stimulation provided by the game, the more money I will spend.	3.80	3.81	3.83	3.43	3.86
The greater the technological innovation of the game, the more money I will spend.	3.70	3.83	3.78	3.35	3.93
The more the game’s materials or storyline align with my preferences, the more money I will spend.	4.00	4.02	3.96	3.67	4.00
The economic investment in the game is a consideration for my spending.	3.20	3.38	3.08	3.24	3.26
The returns from the game’s achievements are a consideration for my spending.	3.20	3.79	3.64	3.53	3.86
The better the art and animation of the game, the more money I will spend.	3.80	3.37	4.00	3.51	3.85
The better the sound effects and music of the game, the more money I will spend.	3.20	3.35	3.41	3.38	3.54
My status within the game is a consideration for my spending.	3.30	3.25	3.30	3.21	3.33
I will spend more money on the game due to my personal preferences.	3.90	3.96	3.98	3.72	4.12
In the future, will you reduce your gaming time for the sake of your health?	3.70	3.90	3.92	4.07	3.86
In the future, will you reduce your gaming time to maintain interpersonal relationships?	3.80	3.86	3.44	3.58	3.42
In the future, will you reduce your gaming time to maintain good financial stability?	3.90	4.08	3.55	3.90	3.77
In the future, will you reduce your gaming time due to work commitments?	4.10	4.13	3.97	3.98	3.96
In the future, will you reduce your gaming time due to insufficient time available for gaming?	4.20	4.04	3.98	3.93	4.09
In the future, will you reduce your gaming time due to changes in your interest?	4.00	4.15	3.77	3.78	4.28

The darker the color, the larger the value; the lighter the color, the smaller the value.

### 3.5 Result

Excluding the respondents who completed the questionnaire in less than 10 min and did not complete the questionnaire, the response was 675. Cluster analysis identified five stages in the subject group: Precontemplation (4.44%), Contemplation (21.33%), Preparation (29.33%), Action (25.78%) and Maintenance (19.11%). Based on demographic characteristics (Table 1), current habits (Table 2), and the proportion of time spent playing games and watching esports live streaming (Table 3), subjects’ consumption status and willingness (Table 4) each cluster was analyzed and statistically compared. The reliability and validity of other parts of the questionnaire were tested. The Cronbach’s *α* was 0.958. The results of Bartlett’s sphericity test and KMO value analysis data were significant at 0.000 (*p* < 0.001). The KMO value was 0.904 (KMO > 0.70), and the collected data could be analyzed by factor analysis. The results of the Confirmatory Factor Analysis (CFA), including the standardized loadings (StdLoading), were presented in Supplementary Table S1. Specifically, for Table 3, CR = 0.4487 and AVE = 0.3611; for Table 4, CR = 0.8554 and AVE = 0.4697; for Table 7, CR = 0.9448 and AVE = 0.5041; for Table 6, CR = 0.9598 and AVE = 0.6484; for Table 9, CR = 0.9622 and AVE = 0.6332; for Table 10, CR = 0.9161 and AVE = 0.4170; and for Table 8, CR = 0.9630 and AVE = 0.5438.

This study conducted a comparative analysis of participants across different consumption stages based on the stages of change model. The following results are presented according to the pre-contemplation, contemplation, preparation, action, and maintenance stages, covering aspects such as demographic characteristics, consumption behaviors, psychological drivers, and barrier factors. Data analysis primarily employed descriptive statistics and mean comparisons. The research findings have been summarized in the corresponding tables, and the following section will analyze the data accordingly.

Reduced the number of people playing games (Table 5); knowledge, attitudes, and beliefs regarding the positive consumption of games (Table 6); knowledge, attitudes, and beliefs regarding the negative consumption of games (Table 7); Knowledge, attitudes, and beliefs regarding the positive aspects of esports live streaming (Table 8); knowledge, attitudes, and beliefs regarding the negative aspects of esports live streaming (Table 9); insights on gaming and observing esports live (Table 10); reasons for discontinuing the watching of esports live broadcasts (Table 11); percentages of reasons for discontinuing the watching of esports live broadcasts (Table 12).

**Table 11:** Reasons for subjects to give up watching live broadcast by esports.

**Type**	**Main concept**	**Initial concept**	**Original statement**
Personal reasons	Wealth	S1: Out of money	No money, no entertainment for me
		S2: Underreturn on	I do not get paid to watch live. Of course I do not
	Time	S3: Busy with work	I’m too busy at work to watch live.
		S4: Busy with housework	I have children, I have to take care of my family, clean the house.
		S5: Busy with study	School is getting busier and less spending on leisure time.
	Interest	S6: Substitutes for interests	I prefer documentaries and rarely watch live
		S7: New goals	I just want to make money right now, so I’ll be more focused on
		S8: Pure dislike	For no other reason, I just do not like watching live
		S9: Family restriction	My wife does not allow me to watch live and wants me to do
	Social relation	S10: Limitation of work	Because of my job, I’m not allowed to watch live games.
		S11: Convergence Effect	My girlfriends like to go shopping now, so I go shopping with
Direct broadcasting room	Consumption	S12: The gradient of spending is small.	Every time I brush gifts, there is no price I can accept, either too
		S13: Paid viewing	If you have to pay for a live stream, I will not watch it.
		S14: High spending limit	Some of the gifts are priced too high, and I would love to use cheap gifts to support my team, but there are no options.
		S15: High data	That’s a lot of traffic. I cannot afford it.
	Atmosphere	S16: Excessive ads	I get tired of watching the commercials in the studio
		S17: Conflicts sparked by comments	The comments are abusive. It’s disgusting
		S18: Fan rivalry	There’s too much black powder with rhythm. It’s a bad vibe.
	Content	S19: Lack of interaction	Now live only watch, anyway, the anchor will interact with me.
		S20: Superficial content	This studio is like a bunch of kindergartners talking.
		S21: Pre-scripted scenario	I feel like there’s a script. I know what story they are going to do
		S22: Lack of innovation	It’s not creative. I’m tired of it.
Player	Image	S23: Ugliness	The studio is ugly. I do not like it.
		S24: Likeness	Every livestream has the same art style. It’s boring.
		S25:sole	Every studio is full of special effects, the image is exactly the
	Ability	S27: Operating level	Not as good as me. I do not even want to watch it
		S28: A weak view of the big picture	His operational skills are too poor
	Stop	S29: Retire from military service	My favorite player retired
		S30: Ban	My favorite player got banned
	Morality	S31: Messy personal life	It pisses me off that my favorite contestant is dating three girls at
		S32: Bully the weak	I hate it when a player maliciously tortures other players at a
		S33: Match-fixing	Ever since my favorite player was exposed as a fake, I hate him.
		S34: Plug-in	He is very good at playing games, but someone exposed that he plays games with plug-ins, and I really hate to open plug-ins.
	Logic	S35: Logical confusion	I do not even know what he’s thinking. It’s a mess.
		S36: Simple logic	His operation is so simple, I can see it when I see it.
		S37: Logical fixation	Playing this way all season, it’s boring.
	Image	S38: Ugliness	The new players are too ugly for my liking
		S39: Likeness	Believe it or not, I stopped watching because a contestant looked
Commentator	Sound line	S40: Shrieks	His voice is awful. It sounds like a duck.
	Frequency	S42: High frequency	The announcer will not stop talking. I wish I could silence him.
	Speech	S45: Personal attack	He said to fans who did not support him: Stupid! Fuck!
	Logic	S46: Logical confusion	I hate a commentator who is confused with logic
		S47: Simple logic	What he said, what I saw, without any thought, was so simple.
Game	Game version	S48: Complex iteration	The major update of the game version is too complicated, causing me to become a “cloud player” who cannot understand the
		S49: High-frequency	The game changes so quickly that I cannot keep up with the game.
		S50: Unbalanced iteration	The version of the game was iterated, and it broke the balance of the game, which made me very disgusted.
	Heat	S51: Heat reduction	The game died, so I moved on to another stream
		S52: Close down	My favorite game went out of business, so I stopped watching

**Table 12:** Proportion analysis of subjects’ reasons for giving up watching esports live broadcast.

**Initial concept**	**Precontemplation**	**Contemplation**	**Preparation**	**Action**	**Maintenance**
Gross amount	33	113	237	171	125
S1: Out of money	1.00	3.00	5.00	4.00	1.00
S2: Underrun on investment	0.00	0.00	1.00	1.00	1.00
S3: Busy with work	5.00	15.00	29.00	22.00	11.00
S4: Busy with housework	3.00	7.00	21.00	16.00	13.00
S5: Busy with study	3.00	8.00	20.00	13.00	11.00
S6: Substitutes for interests	0.00	6.00	8.00	6.00	2.00
S7: New goals	2.00	4.00	8.00	5.00	2.00
S8: Pure dislike	2.00	2.00	9.00	6.00	3.00
S9: Family restriction	1.00	4.00	2.00	1.00	2.00
S10: Limitation of work	0.00	6.00	5.00	5.00	0.00
S11: Convergence Effect	1.00	3.00	1.00	2.00	0.00
S12: The gradient of spending is small.	0.00	1.00	0.00	1.00	0.00
S13: Paid viewing	0.00	0.00	2.00	1.00	0.00
S14: High spending limit	1.00	3.00	5.00	2.00	3.00
S15: High data consumption	0.00	2.00	0.00	1.00	1.00
S16: Excessive ads	0.00	3.00	2.00	2.00	1.00
S17: Conflicts sparked by comments	0.00	1.00	1.00	2.00	2.00
S18: Fan rivalry	0.00	1.00	1.00	2.00	2.00
S19: Lack of interaction	1.00	5.00	9.00	7.00	3.00
S20: Superficial content	2.00	5.00	12.00	7.00	5.00
S21: Pre-scripted scenario	1.00	5.00	11.00	5.00	5.00
S22: Lack of innovation	2.00	5.00	13.00	9.00	7.00
S23: Ugliness	0.00	0.00	3.00	0.00	0.00
S24: Likeness	0.00	0.00	1.00	0.00	1.00
S25: Sole	0.00	1.00	5.00	1.00	1.00
S27: Operating level difference	1.00	1.00	4.00	7.00	4.00
S28: A weak view of the big picture	1.00	1.00	4.00	7.00	4.00
S29: Retire from military service	0.00	1.00	3.00	2.00	2.00
S30: Ban	0.00	2.00	4.00	0.00	2.00
S31: Messy personal life	0.00	2.00	4.00	2.00	2.00
S32: Bully the weak	0.00	1.00	4.00	1.00	2.00
S33: Match-fixing	0.00	1.00	6.00	2.00	3.00
S34: Plug-in	0.00	1.00	5.00	2.00	3.00
S35: Logical confusion	1.00	1.00	2.00	2.00	2.00
S36: Simple logic	1.00	2.00	3.00	4.00	2.00
S37: Logical fixation	1.00	3.00	5.00	5.00	3.00
S38: Ugliness	0.00	0.00	2.00	1.00	2.00
S39: Likeness	0.00	1.00	2.00	1.00	1.00
S40: Shriek	0.00	1.00	2.00	1.00	1.00
S42: High frequency	0.00	0.00	1.00	2.00	3.00
S45: Personal attack	0.00	0.00	1.00	2.00	3.00
S46: Logical confusion	1.00	1.00	2.00	2.00	3.00
S47: Simple logic	0.00	1.00	3.00	2.00	3.00
S48: Complex iteration	1.00	1.00	0.00	1.00	0.00
S49: High-frequency iteration	0.00	1.00	2.00	1.00	1.00
S50: Unbalanced iteration	0.00	1.00	1.00	0.00	0.00
S51: Heat reduction	1.00	0.00	2.00	2.00	1.00
S52: Close down	0.00	0.00	1.00	1.00	1.00

The darker the color, the larger the value; the lighter the color, the smaller the value.

#### 3.5.1 Pre-contemplation stage (4.44%)

Participants in this stage were predominantly young adults aged 18–25 (100%), with the majority holding a bachelor’s (64.5%) or college diploma (29.1%). A higher proportion had received skill-based education (60%), and 80% resided in cities (Table 1). In terms of consumption patterns, a vast majority (72.3%) only played games and did not watch esports live streams. Overall consumption was low, with 40% of participants spending neither on games nor live streams, and another 40% spending only on games (Table 2). Regarding time investment, half of the participants played games for 1–3 h daily, while esports live stream viewing time was generally under 30 min (Table 3). For most players in this stage, the time spent gaming remained unchanged or even increased slightly, with only a small portion beginning to reduce their gaming time (Table 5).

In terms of game consumption intention (Tables 6, 7), participants’ attitudes toward “supporting in-game consumption” (*M* = 3.124) and “not supporting in-game consumption” (*M* = 3.252) were similar in mean scores, indicating an ambiguous consumption inclination. The primary drivers for supporting in-game consumption were psychological needs (*M* = 3.433) and game product features (*M* = 3.240), whereas reasons for opposition centered on physical health (*M* = 3.480), mental health (*M* = 3.300), and interpersonal/social factors (*M* = 3.300). Regarding esports live streaming consumption (Tables 8, 9), attitudes toward supporting (*M* = 3.410) and opposing (*M* = 3.483) also appeared relatively neutral. Key factors supporting consumption included athletes’ physical skills (*M* = 3.566), entertainment and escapism (*M* = 3.400), and knowledge acquisition (*M* = 3.666). In contrast, opposition primarily focused on content format (*M* = 3.560) and ethical concerns (*M* = 3.500).

Drivers of behavioral change (Table 10). Key factors promoting in-game consumption included story selection (*M* = 4.000), art and animation (*M* = 3.900), and personal preferences (*M* = 3.900). The primary reasons for reducing in-game consumption were reduced disposable time (*M* = 4.200), work constraints (*M* = 4.100), and changes in interests (*M* = 4.000). Among the reasons for reducing live-stream viewership, only “busy with work” received considerable recognition (Table 12).

#### 3.5.2 Contemplation stage (21.33%)

Participants in this stage were demographically similar to those in the Pre-contemplation stage in terms of age, education, and residence (Table 1). However, their consumption intentions and behaviors began to show signs of differentiation. In terms of expenditure distribution (Table 2), in-game consumption remained dominant (accounting for 72.9% of spending and 77% of frequency), yet esports live-streaming consumption started to emerge (accounting for 14.5% of spending and 16.6% of frequency). Gaming time was concentrated around 3–5 h (50%), while live-stream viewing was primarily under 30 min per session (Table 3). A significant decline was observed in the time participants spent playing games (Table 5).

In terms of consumption intention (Tables 6–9), participants’ recognition of esports live-streaming consumption increased (*M* = 3.200), particularly evident in the purchase of battle passes (*M* = 3.479) and cultural/creative products (*M* = 3.271). Psychological needs (*M* = 3.486) and product features (*M* = 3.317) remained prominent drivers for supporting in-game consumption, while social needs (*M* = 2.896) were lower. Support for esports live-streaming consumption further strengthened (*M* = 3.847), with the “unpredictability of match outcomes” (*M* = 4.000) becoming a significant attraction. Opposition was concentrated on ethical concerns (*M* = 3.673) and content format (*M* = 3.329).

Regarding drivers of behavioral change (Table 10), game technological innovation (*M* = 3.833), art/animation (*M* = 3.896), and personal preferences (*M* = 3.958) had a significant influence. Beyond time and work constraints, financial pressure (*M* = 4.083) and shifting interests (*M* = 4.146) played a more prominent role in reasons for reducing in-game consumption. Reasons for reducing live-stream viewership became more diverse, including busy household chores, academic pressure, shallow content, and insufficient interaction, among others (Table 12).

#### 3.5.3 Preparation stage (29.33%)

Participants in this stage had a broader age distribution (with 42.4% aged 26–30), primarily held bachelor’s degrees (77.2%), and began to show initial signs of professionalization (Table 1). Regarding expenditure distribution (Table 2), consumption between games and live streaming became more balanced: 45.4% spent more on games, while 36.3% spent more on live streaming. Time investment also showed a diverse distribution for both gaming and live-stream viewing (Table 3). The time spent playing games remained largely consistent with the previous stage (Table 5).

In terms of consumption intention (Tables 6–9), both the frequency (*M* = 3.090) and intention (*M* = 2.970) for esports live-streaming consumption increased significantly. Battle passes (*M* = 3.591) and cultural/creative products (*M* = 3.424) became the primary consumption items. Reasons for supporting live-stream consumption became more defined, with knowledge acquisition (*M* = 4.136), athletes’ physical skills (*M* = 4.086), and drama (*M* = 4.027) receiving strong endorsement. For in-game consumption, psychological needs (*M* = 3.654) and product features (*M* = 3.591) remained the key supporting factors, while social needs (*M* = 3.159) were still relatively weak. Among the reasons for opposition, physical health (*M* = 3.530) and the game product itself (*M* = 3.442) were prominent.

Regarding drivers of behavioral change (Table 10), art/animation (*M* = 4.015) and the sense of game control (*M* = 3.591) had a significant influence. Reasons for reducing consumption still center on time constraints, work pressure, and economic factors. However, barriers to live-stream consumption became more specific, including monotonous content, scripted narratives, and lack of innovation, among others (Table 12).

#### 3.5.4 Action stage (25.78%)

Participants in this stage displayed a higher proportion of those aged 26–30 (48.2%), and most were employed (44.8% being in a working status) (Table 1). Consumption behavior showed a clear tilt toward esports live streaming, with 75.8% of spending and 81% of consumption frequency concentrated on live streaming (Table 2). Viewing time also increased significantly, primarily within the 1–5 h range (Table 3). The time spent playing games saw another substantial decline, and instances of increased gaming time nearly disappeared (Table 5).

In terms of consumption intention (Tables 6–9), the intention for live-streaming consumption strengthened significantly (*M* = 3.350), particularly in tipping streamers/others (*M* = 4.069) and participating in outcome prediction (*M* = 3.621). The mean scores for factors supporting live-stream consumption were all high (overall *M* = 3.95), with athletes’ physical skills, knowledge acquisition, and dramatic performance being the most critical. Support for in-game consumption relatively declined (*M* = 3.221), with emotional connection under social needs receiving the lowest recognition (*M* = 2.879). Reasons opposing in-game consumption remained focused on health and social factors.

Regarding drivers of behavioral change (Table 10), physical health (*M* = 4.069), financial pressure (*M* = 3.931), and reduced time (*M* = 3.931) were the primary reasons for reducing in-game consumption. Reasons for reducing live-stream viewership became more systematic, encompassing content quality, lack of interaction, and rigid commentary styles, with busy work and household chores remaining significant barriers (Table 12).

#### 3.5.5 Maintenance stage (19.11%)

Demographically, participants in the Maintenance stage were similar to those in the Action stage, with the majority concentrated in the 18–30 age range (94.7% combined), holding a bachelor’s degree (81%), residing in cities (82.7%), and nearly 40% having 1–5 years of work experience (Table 1). However, their core consumption pattern underwent a fundamental shift, indicating that the habitual transition from gaming to esports live streaming consumption had stabilized.

Solidification and Transfer of Consumption Behaviors. Data revealed that the overwhelming majority of participants (74.8%) had entered a state of “not playing games, only watching live streams” (Table 2). Consumption focus clearly shifted towards esports live streaming, with 67.4% spending more and 72% consuming more frequently on live streams (Table 3). Correspondingly, gaming time was drastically compressed (nearly half played for less than 30 min per session), while live-stream viewing duration showed a uniform and sustained distribution, spanning from under 30 min to over 3 h, indicating its integration into daily leisure routines (Table 3). Notably, a minimal number of participants showed an increase in gaming time, suggesting a marginal reversal in behavioral change for a small subset (Table 5).

Rationalization and Socialization of Consumption Intention. Although the overall mean scores for both live-stream consumption frequency and intention remained relatively stable (both *M* ≈ 3.0), the internal structure indicated a trend toward rationalization. Among various consumption items, the frequency (*M* = 3.535) and intention (*M* = 3.326) for “tipping streamers/others” were notably prominent, implying that social interaction and support might surpass individual entertainment as key consumption motivations at this stage. In contrast, willingness for direct purchases like cultural and creative products remained moderate (Tables 8, 9).

Stabilization and Reduced Ambivalence in Attitudinal Dimensions. Regarding attitudes toward in-game consumption, the mean difference between support (*M* = 3.218) and opposition (*M* = 3.360) further narrowed, with both being the lowest among all stages, reflecting the significantly diminished importance of gaming in consumers’ lives at this point (Tables 6, 7). For esports live-stream consumption, supportive attitudes, although remaining high (*M* = 3.740), slightly declined compared to the Action stage, while the intensity of opposing attitudes also weakened (*M* = 3.175) (Tables 8, 9). This suggests that consumers in the Maintenance stage have developed a more stable and less conflicted perception of live-stream consumption. Drama (*M* = 4.015) and knowledge acquisition (*M* = 3.852) remained their core value propositions, whereas opposition was primarily focused on relatively mild ethical concerns (*M* = 3.465).

Drivers of Behavior Maintenance and Change. The drivers motivating consumers to maintain or alter their behaviors reached a final equilibrium between “push” and “pull” factors at this stage. For gaming (Table 10), the fundamental shift in interests (*M* = 4.279) and reduction in disposable time (*M* = 4.093) acted as the strongest “push” factors, driving them away from games. Among the potential barriers to live-stream consumption, “busyness” (work, household chores, studies; *Q* = 11–13) emerged as equally prominent factors, revealing that real-life pressures are the primary external constraints on sustained consumption behavior (Table 12). Concurrently, the high recognition of game narrative (*M* = 4.000) and personal preference (*M* = 4.116) corroborated that content quality and alignment with individual interests serve as the key “pull” factors for maintaining long-term engagement.

#### 3.5.6 Results of variance analysis

To determine whether the descriptive differences across stages were statistically supported, one-way ANOVA was conducted on the composite dimensions corresponding to Tables 1–10 (Supplementary Table S2). The results showed that the five TTM stages differed significantly in several key dimensions. Extremely significant differences were found for Table 2 (attitudes toward playing games and watching esports live broadcasts; *F* = 907.1, *p* < 0.001, np2 = 0.942) and Table 3 (current habits; *F* = 9.79, p < 0.001, np2 = 0.151), both indicating large stage effects. Significant differences were also found for Table 5 (changes in the new life stage; *p* = 0.018, np2 = 0.052), Table 8 (support for watching esports live broadcast and consumption; *p* = 0.002, np2 = 0.071), and Table 9 (opposition to watching esports live broadcast and consumption; *p* = 0.028, np2 = 0.047). By contrast, Tables 1, 4, 6, 7, 10 showed no statistically significant differences across TTM stages, indicating that the descriptive variations in these dimensions should be interpreted more cautiously.

Post-hoc tests were further conducted only for the dimensions with significant ANOVA results (Supplementary Table S3). For Table 2, the maintenance stage differed significantly from all other stages, and the action stage was significantly higher than the pre-contemplation, contemplation, and preparation stages. This confirms that the most pronounced stage difference occurs in users’ basic orientation toward gameplay versus live-streaming. For Table 3, the contemplation stage was significantly lower than the preparation, action, and maintenance stages, indicating that actual habits become more developed after users move beyond mere intention. For Tables 8, 9, significant differences were mainly concentrated between later stages (action and maintenance) and earlier stages (pre-contemplation and contemplation), suggesting that support for live-streaming consumption increases and opposition to live-streaming consumption weakens as users advance through the TTM stages.

## 4 Discussion

The findings indicate that participants’ gaming-related consumption does not shift in a simple linear way from playing games to watching esports live streaming. Instead, the five TTM stages reveal different consumption profiles within the broader gaming ecosystem. From the precontemplation stage to the action stage, participants showed a gradual shift from game-centered engagement toward live-streaming-centered engagement. Their preferences, consumption frequency, and consumption amount increasingly favored esports live streaming. However, this tendency weakened in the maintenance stage, suggesting that behavioral substitution may not be permanent or absolute. Rather, users may reorganize their engagement by combining gameplay, viewing, learning, and symbolic consumption.

The ANOVA results for Tables 2, 3 provide statistical support for this pattern. Significant differences were found across the five TTM stages, especially between the action and maintenance stages and the earlier stages. This suggests that the most visible behavioral shift occurs after users move beyond intention and begin to adopt new consumption habits. Participants in the preparation, action, and maintenance stages had more distinct patterns of game and live-streaming consumption than those in the contemplation stage. Therefore, the TTM stages are useful for describing different behavioral positions, but they should be understood as cross-sectional stage-based profiles rather than direct evidence of longitudinal behavioral change.

The demographic pattern further helps explain this shift. Participants in the early stages were mainly younger university students, who still had more time and opportunity to play games. In the middle stages, the proportion of older students and early-career workers increased, and their gaming time declined while live-streaming consumption became more prominent. This indicates that the player-to-viewer shift is closely related to changes in life rhythm, available time, and daily responsibility. In the maintenance stage, some participants showed a partial return to gameplay, possibly because returning to university life or having a slower life rhythm restored their disposable time. This suggests that behavioral substitution is reversible and may fluctuate according to life conditions.

In terms of motivations for game consumption, Table 6 shows that psychological needs, game product features, and social needs were all recognized by participants. Among them, personalized psychological needs were the most important reason supporting gameplay and in-game consumption. This finding is consistent with gaming motivation research, which emphasizes that players engage with games to satisfy needs such as challenge, autonomy, competence, entertainment, immersion, escapism, self-expression, and self-esteem. However, the ANOVA results showed no significant differences across TTM stages for Tables 6, 7. This means that these motivations and concerns are shared across users at different stages. What changes across stages is not users’ basic evaluation of games, but their behavioral position between gameplay and live-streaming consumption.

The reasons for reducing gameplay also show that users do not leave games only because of external pressure. They are also influenced by changes in personal interest, attitudes, health concerns, game quality, moral issues, and social experience. Problems such as insufficient challenge, lack of creativity, weak operability, negative social interaction, verbal abuse, harassment, sleep disturbance, and visual fatigue may weaken users’ willingness to continue playing. These factors suggest that the decline in gameplay is driven by both product-level dissatisfaction and psychological fatigue.

Compared with game-related evaluations, attitudes toward esports live streaming showed clearer stage differences. The ANOVA results for Tables 8, 9 showed significant differences across TTM stages. Later-stage participants, especially those in the action and maintenance stages, showed stronger recognition of live-streaming consumption and lower resistance to watching. This indicates that live streaming gradually becomes a legitimate and meaningful form of gaming-related participation. Users do not merely stop playing games; they reconstruct their value perception through watching, learning, emotional involvement, and indirect achievement.

The motivations for watching esports live streaming include entertainment, avoidance, knowledge acquisition, conformity, drama, physical convenience, mental relaxation, and reduced operational burden. Among these factors, the uncertainty and drama of competition, the ability to learn strategies and skills, and the convenience of watching are especially important. These elements allow viewers to obtain value without the time, energy, and skill investment required by direct gameplay. Therefore, the value of gaming consumption shifts from “what I can do” to “what I can see, learn, feel, and follow.”

At the same time, live-streaming consumption also faces barriers. Poor player performance, weak commentary, unattractive visual style, shallow information, lack of interaction, excessive advertising, negative comments, moral issues, high traffic requirements, unstable signals, and unsuitable broadcast times may reduce users’ willingness to watch. These factors show that esports live streaming is not a passive replacement for gameplay, but a new consumption scene with its own quality requirements. Viewers still evaluate the content according to entertainment value, emotional experience, information depth, social atmosphere, and convenience.

Tables 11, 12 further show that reduced disposable time is a key condition behind behavioral change. Busy work, family responsibility, learning pressure, financial limitation, and workplace restrictions all reduce users’ ability to continue playing games. Compared with gameplay, watching esports live streaming requires less time, less physical effort, and lower operational involvement. This makes live streaming a more flexible form of gaming-related consumption for users whose life rhythms have become more constrained. However, personal attitudes, changing interests, new life goals, and alternative leisure activities also affect whether users continue watching live streams. Thus, behavioral substitution is shaped by both external constraints and internal preference changes.

Overall, the findings suggest that the shift from player to viewer should not be understood as a complete replacement of gameplay by spectatorship. It is better understood as a stage-conditioned reorganization of gaming engagement. Some users may reduce gameplay and turn to live streaming; some may combine playing and watching; others may move back and forth between the two depending on time, interest, life stage, and content quality. The contribution of this study is therefore to show how gaming consumption changes from direct operational participation to a more flexible structure of viewing, learning, emotional involvement, and symbolic consumption. This extends the TTM framework from individual behavior change to the study of digital media consumption and provides a clearer explanation of how users reposition themselves within the gaming ecosystem.

## 5 Research conclusions and practical recommendations

### 5.1 Research conclusions

Based on the transtheoretical Model (TTM), this study analyzed the behavioral transition process of China’s game users from “players” to “audiences” in phases, and drew the following core conclusions:

Firstly, behavioral substitution follows a distinct nonlinear evolutionary path that is closely tied to life stages. Data confirms that the five-phase model, from “unintentional” to “sustained” usage, effectively describes user migration. This process is not linear; rather, it exhibits pronounced peaks in consumption and attention during the “determination” and “action” phases, subsequently tapering off in a measured decline during the “sustained” phase. The study found that age was significantly positively correlated with the stage of behavior, and that ageing (particularly the transition from student to working adult) was a key external driver of this substitution process. The most notable shift across these phases arises from profound life structure changes (e.g., academic advancement or employment) that reduce disposable time. This extends beyond mere shifts in interest, revealing the underlying logic of real-world resource constraints that drive behavioral substitution.

Secondly, the psychological decision-making mechanism that drives substitution operates through a dynamic equilibrium of “motivation,” “attractiveness,” and “resistance.” This study demonstrates that substitution is not driven by a single factor. Real-time constraints, work demands, and awareness of health risks (e.g., vision and sleep) all serve as driving forces for players to substitute behaviors. The substitutional satisfaction derived from live streaming’s “knowledge acquisition” value and “the ornamental value of high-level esports competition (drama and unpredictability)” transcends mere “entertainment and escapism,” becoming a compelling incentive. Dynamic resistance manifests as follows: initial resistance primarily stems from game immersion and existing social bonds, but as substitution deepens, live streaming offers a new sense of community belonging (conformity), partially fulfilling old social needs and demonstrating the transformability of resistance.

Thirdly, consumer behavior and attitudes demonstrate a dual dynamic characterized by “idealization” and “rational adjustment.” Research indicates that users exhibit peak willingness and anticipation for live-streaming consumption during the “intention” and “determination” phases, reflecting strong idealization. After entering the ‘action’ and ‘maintenance’ phases, although consumer behavior has firmly shifted to live streaming, both consumption frequency and some supportive attitudes have declined from their peak rationality. This suggests that what ultimately sustains alternative behaviors is not the initial novelty, but the sustained recognition of the core values of live streaming (knowledge and entertainment value) and the persistent avoidance of its negative aspects (such as health risks and ethical concerns). The user’s actual behavior migration (consumption amount, time allocation) is systematically ahead of its complete identity transformation.

Fourth, the fundamental distinction between the “support-opposition” logic underlying gaming and live-streaming consumption reveals deeper motivations for behavioral substitution. Gaming consumption is motivated by “intrinsic self-actualization” (psychological needs, personalization, and a sense of control), whereas live-streaming consumption stems from “extrinsic learning and appreciation” (knowledge acquisition and admiration of athletes’ skills). Opposition to gaming consumption often arises from “avoiding personal harm” (health, time, money, and social aspects), whereas opposition to live-streaming consumption typically stems from “dissatisfaction with external content quality” (superficial content, lack of innovation, and ethical concerns). This distinction demonstrates that behavioral substitution is not merely a shift in activities but a reconfiguration of value acquisition logic, from “internal control-seeking” to “external connection-based appreciation and learning.”

### 5.2 Practical recommendations

Based on the stage-based findings, platforms, game companies, and esports live-streaming services should adopt differentiated engagement strategies for users at different TTM stages.

First, for users in the precontemplation and contemplation stages, the key task is to build awareness and reduce the psychological distance between gameplay and esports live streaming. These users still mainly identify as players and have not yet formed a stable intention to shift their consumption behavior. Therefore, game companies and streaming platforms can embed low-threshold live-streaming entrances into games, such as recommended match highlights, streamer tutorials, short tactical clips, and event reminders. In-game rewards linked to watching esports content can also help users understand that live streaming is not separate from gameplay, but can enhance their game knowledge, skills, and community participation. At this stage, the main strategy is not to push users to abandon gameplay, but to help them recognize live streaming as an extension of game-related engagement.

Second, for users in the preparation and action stages, the key task is to convert behavioral intention into stable viewing and consumption behavior. These users have already shown stronger interest in esports live streaming and are more sensitive to content quality, interaction, and consumption value. Platforms should therefore strengthen the core attractions of live streaming, including high-level competition, dramatic uncertainty, professional commentary, skill learning, and interactive participation. At the same time, diversified consumption options should be provided, such as virtual gifts, battle passes, memberships, prediction activities, cultural and creative products, and team-related merchandise. Since users in these stages still compare the value of gameplay and live streaming, platforms should emphasize what viewers can gain through watching, including tactical knowledge, emotional involvement, social interaction, and symbolic support for players or teams.

Third, for users in the maintenance stage, the key task is to sustain long-term engagement and prevent decline after novelty fades. These users have already formed relatively stable live-streaming habits, but their consumption becomes more rational and may fluctuate with time pressure, work, family responsibilities, and changing interests. Therefore, platforms should provide shorter, more frequent, and more flexible content formats, such as condensed match highlights, replay packages, audio-based commentary, mobile-friendly viewing modes, and personalized recommendations. Community-building is also important at this stage. Online forums, fan groups, offline events, team communities, and streamer-centered interaction can strengthen users’ sense of belonging and social recognition. Meanwhile, platforms should improve ethical governance by reducing toxic comments, false advertising, cheating, match-fixing, and uncivilized streamer behavior, because a fair and healthy live-streaming environment is essential for maintaining long-term trust and sustainable consumption.

Overall, the transformation from player to viewer requires coordinated efforts from game developers, esports organizations, streamers, platforms, and regulators. The goal is not simply to replace gameplay with live streaming, but to build a more flexible gaming ecosystem in which users can shift among playing, watching, learning, interacting, and consuming according to their life stage, available time, and psychological needs.

### 5.3 Research limitations and future prospects

This study has certain limitations. Firstly, cross-sectional data are difficult to infer causality and individual dynamic trajectory strictly. Then, the sample is concentrated on young Chinese users, and the cross-cultural generalizability of the conclusions needs to be tested. Finally, relying on self-reported data may lead to deflection.

Future research could explore the following directions: (1) Longitudinal tracking design: Track the same users over time to accurately map the trigger points and trajectories of changes. (2) Cross-cultural comparison: Investigating the similarities and differences in behavioral substitution mechanisms across various socio-cultural contexts. (3) Multimodal integration: Integrating objective behavioral data (backend viewing/consumption logs) with physiological measurements (eye tracking, heart rate) to thoroughly uncover the cognitive and emotional engagement mechanisms of “spectator gaze.” (4) Reentry mechanism exploration: Examining psychological and contextual factors that contribute to regression from the “sustained phase” to earlier developmental stages, thereby refining nonlinear behavioral models. (5) Regarding the study’s findings, some participants proposed “pure voice commentary formats for game event live broadcasts” as a potential future research direction. This implies not only behavioral substitution from “player” to “spectator” but also from “player” to “audience,” and even from “spectator” to “audience.” Although these aspects cannot be fully explored in this paper, they offer conceptual insights for game companies to develop new services and growth opportunities, as well as to advance related research.

In summary, the transition from gamers to audiences represents an active “media consumption strategy restructuring” undertaken by players to optimize personal well-being under real-world resource constraints. Understanding the phased patterns and psychological mechanisms of this dynamic process holds significant importance for academic research to deepen digital consumption behavior theories, as well as for industry practices to achieve refined user operations and lifecycle management in existing market competition.

## Data Availability

The original contributions presented in the study are included in the article/Supplementary material, further inquiries can be directed to the corresponding author.
