# Supplementary material for: From “player” to “viewer”: a survey on the willingness of video game players to alter their game consumption behavior
Source: Front Psychol. 2026 Jul 3;17:1807531. doi: 10.3389/fpsyg.2026.1807531 (PMC13382615; doi:10.3389/fpsyg.2026.1807531)
Supplement: Supplementary file 1 [file Table_1.docx]

Table S1 Confirmatory Factor Analysis (CFA) and Standardized Loadings

| Table | Table Name Abbreviation | Item | Loading | StdLoading | CR | AVE | StdLoading_sq | ErrorVar(1-StdL^2) |
| --- | --- | --- | --- | --- | --- | --- | --- | --- |
| Table 7 |  |  |  |  | 0.9448 | 0.5041 |  |  |
| Table 3 |  |  |  |  | 0.4487 | 0.3611 |  |  |
| Table 5 |  |  |  |  | 1.0000 | 1.0000 |  |  |
| Table 6 |  |  |  |  | 0.9598 | 0.6484 |  |  |
| Table 9 |  |  |  |  | 0.9623 | 0.6333 |  |  |
| Table 4 |  |  |  |  | 0.8554 | 0.4697 |  |  |
| Table 2 |  |  |  |  | 1.0000 | 1.0000 |  |  |
| Table 10 |  |  |  |  | 0.9161 | 0.4170 |  |  |
| Table 8 |  |  |  |  | 0.9630 | 0.5438 |  |  |
| Table 7 | AGC | AGC1 | 1.0000 | 0.6401 | 0.9448 | 0.5041 | 0.4097 | 0.5903 |
| Table 7 | AGC | AGC10 | 0.8421 | 0.6726 | 0.9448 | 0.5041 | 0.4524 | 0.5476 |
| Table 7 | AGC | AGC11 | 0.9682 | 0.7188 | 0.9448 | 0.5041 | 0.5167 | 0.4833 |
| Table 7 | AGC | AGC12 | 0.8050 | 0.6472 | 0.9448 | 0.5041 | 0.4189 | 0.5811 |
| Table 7 | AGC | AGC13 | 1.0276 | 0.7851 | 0.9448 | 0.5041 | 0.6164 | 0.3836 |
| Table 7 | AGC | AGC14 | 1.0665 | 0.7466 | 0.9448 | 0.5041 | 0.5574 | 0.4426 |
| Table 7 | AGC | AGC15 | 1.1996 | 0.7872 | 0.9448 | 0.5041 | 0.6196 | 0.3804 |
| Table 7 | AGC | AGC16 | 1.0669 | 0.7603 | 0.9448 | 0.5041 | 0.5781 | 0.4219 |
| Table 7 | AGC | AGC17 | 1.0561 | 0.7163 | 0.9448 | 0.5041 | 0.5131 | 0.4869 |
| Table 7 | AGC | AGC2 | 0.9623 | 0.6452 | 0.9448 | 0.5041 | 0.4163 | 0.5837 |
| Table 7 | AGC | AGC3 | 1.0453 | 0.6966 | 0.9448 | 0.5041 | 0.4853 | 0.5147 |
| Table 7 | AGC | AGC4 | 1.0562 | 0.7219 | 0.9448 | 0.5041 | 0.5211 | 0.4789 |
| Table 7 | AGC | AGC5 | 1.0181 | 0.7414 | 0.9448 | 0.5041 | 0.5497 | 0.4503 |
| Table 7 | AGC | AGC6 | 1.1698 | 0.7886 | 0.9448 | 0.5041 | 0.6218 | 0.3782 |
| Table 7 | AGC | AGC7 | 0.7436 | 0.5209 | 0.9448 | 0.5041 | 0.2714 | 0.7286 |
| Table 7 | AGC | AGC8 | 1.1395 | 0.7718 | 0.9448 | 0.5041 | 0.5957 | 0.4043 |
| Table 7 | AGC | AGC9 | 0.9859 | 0.6526 | 0.9448 | 0.5041 | 0.4259 | 0.5741 |
| Table 3 | CHS | CHS1 | 1.0000 | 0.7473 | 0.4487 | 0.3611 | 0.5585 | 0.4415 |
| Table 3 | CHS | CHS2 | 0.8563 | 0.8597 | 0.4487 | 0.3611 | 0.7392 | 0.2608 |
| Table 3 | CHS | CHS3 | -0.6033 | -0.3406 | 0.4487 | 0.3611 | 0.1160 | 0.8840 |
| Table 3 | CHS | CHS4 | 0.3692 | 0.1758 | 0.4487 | 0.3611 | 0.0309 | 0.9691 |
| Table 5 | CS | CS1 | 1.0000 | 1.0000 | 1.0000 | 1.0000 | 1.0000 | 0.0000 |
| Table 6 | KAB | KAB1 | 1.0000 | 0.8263 | 0.9598 | 0.6484 | 0.6827 | 0.3173 |
| Table 6 | KAB | KAB10 | 1.0126 | 0.8197 | 0.9598 | 0.6484 | 0.6719 | 0.3281 |
| Table 6 | KAB | KAB11 | 0.9330 | 0.7897 | 0.9598 | 0.6484 | 0.6236 | 0.3764 |
| Table 6 | KAB | KAB12 | 0.9953 | 0.7430 | 0.9598 | 0.6484 | 0.5521 | 0.4479 |
| Table 6 | KAB | KAB13 | 0.9244 | 0.6654 | 0.9598 | 0.6484 | 0.4428 | 0.5572 |
| Table 6 | KAB | KAB2 | 1.0140 | 0.8416 | 0.9598 | 0.6484 | 0.7082 | 0.2918 |
| Table 6 | KAB | KAB3 | 1.0107 | 0.8221 | 0.9598 | 0.6484 | 0.6759 | 0.3241 |
| Table 6 | KAB | KAB4 | 0.9985 | 0.8010 | 0.9598 | 0.6484 | 0.6416 | 0.3584 |
| Table 6 | KAB | KAB5 | 1.0419 | 0.8459 | 0.9598 | 0.6484 | 0.7155 | 0.2845 |
| Table 6 | KAB | KAB6 | 1.1199 | 0.8204 | 0.9598 | 0.6484 | 0.6731 | 0.3269 |
| Table 6 | KAB | KAB7 | 1.0062 | 0.8216 | 0.9598 | 0.6484 | 0.6750 | 0.3250 |
| Table 6 | KAB | KAB8 | 0.9485 | 0.8055 | 0.9598 | 0.6484 | 0.6488 | 0.3512 |
| Table 6 | KAB | KAB9 | 1.0711 | 0.8476 | 0.9598 | 0.6484 | 0.7185 | 0.2815 |
| Table 9 | OWC | OWC1 | 1.0000 | 0.7796 | 0.9623 | 0.6333 | 0.6078 | 0.3922 |
| Table 9 | OWC | OWC10 | 0.6618 | 0.5446 | 0.9623 | 0.6333 | 0.2966 | 0.7034 |
| Table 9 | OWC | OWC11 | 0.8117 | 0.6389 | 0.9623 | 0.6333 | 0.4082 | 0.5918 |
| Table 9 | OWC | OWC12 | 1.0737 | 0.8568 | 0.9623 | 0.6333 | 0.7341 | 0.2659 |
| Table 9 | OWC | OWC13 | 1.1128 | 0.8731 | 0.9623 | 0.6333 | 0.7623 | 0.2377 |
| Table 9 | OWC | OWC14 | 1.1604 | 0.8738 | 0.9623 | 0.6333 | 0.7636 | 0.2364 |
| Table 9 | OWC | OWC15 | 1.0822 | 0.8469 | 0.9623 | 0.6333 | 0.7172 | 0.2828 |
| Table 9 | OWC | OWC2 | 1.0555 | 0.8197 | 0.9623 | 0.6333 | 0.6718 | 0.3282 |
| Table 9 | OWC | OWC3 | 1.0601 | 0.7956 | 0.9623 | 0.6333 | 0.6330 | 0.3670 |
| Table 9 | OWC | OWC4 | 1.0756 | 0.8218 | 0.9623 | 0.6333 | 0.6753 | 0.3247 |
| Table 9 | OWC | OWC5 | 1.0345 | 0.8235 | 0.9623 | 0.6333 | 0.6781 | 0.3219 |
| Table 9 | OWC | OWC6 | 1.0914 | 0.8632 | 0.9623 | 0.6333 | 0.7451 | 0.2549 |
| Table 9 | OWC | OWC7 | 1.2206 | 0.8816 | 0.9623 | 0.6333 | 0.7773 | 0.2227 |
| Table 9 | OWC | OWC8 | 1.0259 | 0.7947 | 0.9623 | 0.6333 | 0.6315 | 0.3685 |
| Table 9 | OWC | OWC9 | 0.8207 | 0.6302 | 0.9623 | 0.6333 | 0.3972 | 0.6028 |
| Table 4 | SCW | SCW1 | 1.0000 | 0.6114 | 0.8554 | 0.4697 | 0.3738 | 0.6262 |
| Table 4 | SCW | SCW10 | 1.1231 | 0.6956 | 0.8554 | 0.4697 | 0.4838 | 0.5162 |
| Table 4 | SCW | SCW2 | 1.1271 | 0.7054 | 0.8554 | 0.4697 | 0.4976 | 0.5024 |
| Table 4 | SCW | SCW3 | 1.2412 | 0.7208 | 0.8554 | 0.4697 | 0.5195 | 0.4805 |
| Table 4 | SCW | SCW4 | 1.1214 | 0.6841 | 0.8554 | 0.4697 | 0.4680 | 0.5320 |
| Table 4 | SCW | SCW5 | 0.8829 | 0.5283 | 0.8554 | 0.4697 | 0.2791 | 0.7209 |
| Table 4 | SCW | SCW6 | 1.2355 | 0.7441 | 0.8554 | 0.4697 | 0.5536 | 0.4464 |
| Table 4 | SCW | SCW7 | 1.1405 | 0.6682 | 0.8554 | 0.4697 | 0.4465 | 0.5535 |
| Table 4 | SCW | SCW8 | 1.2692 | 0.7445 | 0.8554 | 0.4697 | 0.5542 | 0.4458 |
| Table 4 | SCW | SCW9 | 1.1567 | 0.7214 | 0.8554 | 0.4697 | 0.5205 | 0.4795 |
| Table 2 | SGW | SGW1 | 1.0000 | 1.0000 | 1.0000 | 1.0000 | 1.0000 | 0.0000 |
| Table 10 | SP | SP1 | 1.0000 | 0.7210 | 0.9161 | 0.4170 | 0.5198 | 0.4802 |
| Table 10 | SP | SP10 | 0.9423 | 0.7492 | 0.9161 | 0.4170 | 0.5613 | 0.4387 |
| Table 10 | SP | SP11 | 0.9071 | 0.7233 | 0.9161 | 0.4170 | 0.5231 | 0.4769 |
| Table 10 | SP | SP12 | 1.0892 | 0.7520 | 0.9161 | 0.4170 | 0.5655 | 0.4345 |
| Table 10 | SP | SP13 | 0.8256 | 0.6412 | 0.9161 | 0.4170 | 0.4112 | 0.5888 |
| Table 10 | SP | SP14 | 0.1844 | 0.1530 | 0.9161 | 0.4170 | 0.0234 | 0.9766 |
| Table 10 | SP | SP15 | 0.3135 | 0.2117 | 0.9161 | 0.4170 | 0.0448 | 0.9552 |
| Table 10 | SP | SP16 | 0.1614 | 0.1257 | 0.9161 | 0.4170 | 0.0158 | 0.9842 |
| Table 10 | SP | SP17 | 0.1835 | 0.1498 | 0.9161 | 0.4170 | 0.0224 | 0.9776 |
| Table 10 | SP | SP18 | 0.2040 | 0.1705 | 0.9161 | 0.4170 | 0.0291 | 0.9709 |
| Table 10 | SP | SP19 | 0.2330 | 0.1840 | 0.9161 | 0.4170 | 0.0339 | 0.9661 |
| Table 10 | SP | SP2 | 1.1252 | 0.8383 | 0.9161 | 0.4170 | 0.7028 | 0.2972 |
| Table 10 | SP | SP3 | 1.1504 | 0.8346 | 0.9161 | 0.4170 | 0.6965 | 0.3035 |
| Table 10 | SP | SP4 | 1.1419 | 0.8532 | 0.9161 | 0.4170 | 0.7280 | 0.2720 |
| Table 10 | SP | SP5 | 1.1232 | 0.8645 | 0.9161 | 0.4170 | 0.7473 | 0.2527 |
| Table 10 | SP | SP6 | 1.0419 | 0.8359 | 0.9161 | 0.4170 | 0.6988 | 0.3012 |
| Table 10 | SP | SP7 | 0.9233 | 0.7468 | 0.9161 | 0.4170 | 0.5577 | 0.4423 |
| Table 10 | SP | SP8 | 1.0395 | 0.7059 | 0.9161 | 0.4170 | 0.4983 | 0.5017 |
| Table 10 | SP | SP9 | 1.0437 | 0.7368 | 0.9161 | 0.4170 | 0.5429 | 0.4571 |
| Table 8 | SWC | SWC1 | 1.0000 | 0.7706 | 0.9630 | 0.5438 | 0.5938 | 0.4062 |
| Table 8 | SWC | SWC10 | 0.8895 | 0.6486 | 0.9630 | 0.5438 | 0.4207 | 0.5793 |
| Table 8 | SWC | SWC11 | 0.9399 | 0.7241 | 0.9630 | 0.5438 | 0.5243 | 0.4757 |
| Table 8 | SWC | SWC12 | 0.9190 | 0.7356 | 0.9630 | 0.5438 | 0.5411 | 0.4589 |
| Table 8 | SWC | SWC13 | 1.1095 | 0.7775 | 0.9630 | 0.5438 | 0.6045 | 0.3955 |
| Table 8 | SWC | SWC14 | 1.1568 | 0.7744 | 0.9630 | 0.5438 | 0.5997 | 0.4003 |
| Table 8 | SWC | SWC15 | 1.2023 | 0.7726 | 0.9630 | 0.5438 | 0.5969 | 0.4031 |
| Table 8 | SWC | SWC16 | 1.1351 | 0.7625 | 0.9630 | 0.5438 | 0.5815 | 0.4185 |
| Table 8 | SWC | SWC17 | 1.2077 | 0.7930 | 0.9630 | 0.5438 | 0.6289 | 0.3711 |
| Table 8 | SWC | SWC18 | 1.2155 | 0.8094 | 0.9630 | 0.5438 | 0.6552 | 0.3448 |
| Table 8 | SWC | SWC19 | 1.1680 | 0.7780 | 0.9630 | 0.5438 | 0.6054 | 0.3946 |
| Table 8 | SWC | SWC2 | 1.0038 | 0.7870 | 0.9630 | 0.5438 | 0.6193 | 0.3807 |
| Table 8 | SWC | SWC20 | 0.9467 | 0.7320 | 0.9630 | 0.5438 | 0.5358 | 0.4642 |
| Table 8 | SWC | SWC21 | 0.8867 | 0.6902 | 0.9630 | 0.5438 | 0.4764 | 0.5236 |
| Table 8 | SWC | SWC22 | 0.9186 | 0.6736 | 0.9630 | 0.5438 | 0.4538 | 0.5462 |
| Table 8 | SWC | SWC3 | 0.9688 | 0.7408 | 0.9630 | 0.5438 | 0.5488 | 0.4512 |
| Table 8 | SWC | SWC4 | 0.7958 | 0.5356 | 0.9630 | 0.5438 | 0.2868 | 0.7132 |
| Table 8 | SWC | SWC5 | 0.9498 | 0.7456 | 0.9630 | 0.5438 | 0.5559 | 0.4441 |
| Table 8 | SWC | SWC6 | 0.9395 | 0.7608 | 0.9630 | 0.5438 | 0.5788 | 0.4212 |
| Table 8 | SWC | SWC7 | 0.9489 | 0.7692 | 0.9630 | 0.5438 | 0.5917 | 0.4083 |
| Table 8 | SWC | SWC8 | 0.9760 | 0.7299 | 0.9630 | 0.5438 | 0.5328 | 0.4672 |
| Table 8 | SWC | SWC9 | 0.9499 | 0.6566 | 0.9630 | 0.5438 | 0.4312 | 0.5688 |

Table S2 ANOVA Results

| Variable | F | df_num | df_den | p-value | np2 | Levene_p | Equal_Var |
| --- | --- | --- | --- | --- | --- | --- | --- |
| Table1 | 1.51491985 | 4 | 220 | 0.198791924 | 0.026805662 | 0.316291237 | TRUE |
| Table2 | 907.1088857 | 4 | 220 | 2.0194E-135 | 0.942833913 | 1.28671E-09 | FALSE |
| Table3 | 9.79163881 | 4 | 220 | 2.62366E-07 | 0.151125037 | 0.010150392 | FALSE |
| Table4 | 0.662102344 | 4 | 220 | 0.618956773 | 0.011895029 | 0.674103395 | TRUE |
| Table5 | 3.035315302 | 4 | 220 | 0.018333383 | 0.052301177 | 0.93999531 | TRUE |
| Table6 | 1.201318846 | 4 | 220 | 0.311165091 | 0.021375279 | 0.031900736 | FALSE |
| Table7 | 0.132428244 | 4 | 220 | 0.970365407 | 0.002402003 | 0.166068405 | TRUE |
| Table8 | 4.191804572 | 4 | 220 | 0.002723367 | 0.070817313 | 0.17917351 | TRUE |
| Table9 | 2.760023104 | 4 | 220 | 0.02865512 | 0.047784314 | 0.422119108 | TRUE |
| Table10 | 0.562433581 | 4 | 220 | 0.690169257 | 0.010122551 | 0.99853237 | TRUE |

Table S3 PostHoc Results

| A | B | mean_A | mean_B | diff | se | T | df | pval | hedges | Test | Variable | p_tukey |
| --- | --- | --- | --- | --- | --- | --- | --- | --- | --- | --- | --- | --- |
| 1 | 2 | 5 | 2 | 3 | 0 | inf |  |  | inf | Games-Howell | Table2 |  |
| 1 | 3 | 5 | 1 | 4 | 0 | inf |  |  | inf | Games-Howell | Table2 |  |
| 1 | 4 | 5 | 2.965517241 | 2.034482759 | 0.034482759 | 59 | 57 | 0 | 8.241194315 | Games-Howell | Table2 |  |
| 1 | 5 | 5 | 5.488372093 | -0.488372093 | 0.134650579 | -3.626958718 | 42 | 0.006552629 | -0.60048586 | Games-Howell | Table2 |  |
| 2 | 3 | 2 | 1 | 1 | 0 | inf |  |  | inf | Games-Howell | Table2 |  |
| 2 | 4 | 2 | 2.965517241 | -0.965517241 | 0.034482759 | -28 | 57 | 0 | -4.93028921 | Games-Howell | Table2 |  |
| 2 | 5 | 2 | 5.488372093 | -3.488372093 | 0.134650579 | -25.90684799 | 42 | 0 | -5.702496372 | Games-Howell | Table2 |  |
| 3 | 4 | 1 | 2.965517241 | -1.965517241 | 0.034482759 | -57 | 57 | 0 | -10.88227626 | Games-Howell | Table2 |  |
| 3 | 5 | 1 | 5.488372093 | -4.488372093 | 0.134650579 | -33.33347775 | 42 | 0 | -8.056596702 | Games-Howell | Table2 |  |
| 4 | 5 | 2.965517241 | 5.488372093 | -2.522854852 | 0.138995824 | -18.15058016 | 47.53890879 | 9.32587E-15 | -4.113509139 | Games-Howell | Table2 |  |
| 1 | 2 | 2.125 | 1.84375 | 0.28125 | 0.165948976 | 1.694798049 | 11.84060629 | 0.472251902 | 0.649043342 | Games-Howell | Table3 |  |
| 1 | 3 | 2.125 | 2.306818182 | -0.181818182 | 0.174165129 | -1.043941359 | 14.28687006 | 0.831053424 | -0.285010709 | Games-Howell | Table3 |  |
| 1 | 4 | 2.125 | 2.448275862 | -0.323275862 | 0.167203682 | -1.933425498 | 12.20131427 | 0.351146771 | -0.66212512 | Games-Howell | Table3 |  |
| 1 | 5 | 2.125 | 2.23255814 | -0.10755814 | 0.167951584 | -0.640411582 | 12.39218502 | 0.965324206 | -0.241363058 | Games-Howell | Table3 |  |
| 2 | 3 | 1.84375 | 2.306818182 | -0.463068182 | 0.099779197 | -4.640929142 | 110.3694428 | 9.28146E-05 | -0.817822016 | Games-Howell | Table3 |  |
| 2 | 4 | 1.84375 | 2.448275862 | -0.604525862 | 0.087060135 | -6.943773549 | 103.8238118 | 3.40943E-09 | -1.326208314 | Games-Howell | Table3 |  |
| 2 | 5 | 1.84375 | 2.23255814 | -0.38880814 | 0.088488024 | -4.393906905 | 87.24570411 | 0.000296109 | -0.916366737 | Games-Howell | Table3 |  |
| 3 | 4 | 2.306818182 | 2.448275862 | -0.14145768 | 0.101852329 | -1.388850722 | 118.8100976 | 0.635992016 | -0.243794727 | Games-Howell | Table3 |  |
| 3 | 5 | 2.306818182 | 2.23255814 | 0.074260042 | 0.103075507 | 0.720443143 | 106.9751878 | 0.951368012 | 0.12888522 | Games-Howell | Table3 |  |
| 4 | 5 | 2.448275862 | 2.23255814 | 0.215717723 | 0.090819266 | 2.375241852 | 95.75699141 | 0.131083624 | 0.465992623 | Games-Howell | Table3 |  |
| 1 | 2 | 2.7 | 2.020833333 | 0.679166667 | 0.325057711 | 2.089372574 |  |  | 0.663665714 | Tukey HSD | Table5 | 0.228351067 |
| 1 | 3 | 2.7 | 2.045454545 | 0.654545455 | 0.317323272 | 2.062708641 |  |  | 0.661604691 | Tukey HSD | Table5 | 0.240151709 |
| 1 | 4 | 2.7 | 1.706896552 | 0.993103448 | 0.320189845 | 3.101608194 |  |  | 1.200825448 | Tukey HSD | Table5 | 0.018339137 |
| 1 | 5 | 2.7 | 2.11627907 | 0.58372093 | 0.328299939 | 1.778011084 |  |  | 0.615536116 | Tukey HSD | Table5 | 0.389035379 |
| 2 | 3 | 2.020833333 | 2.045454545 | -0.024621212 | 0.177389102 | -0.138797772 |  |  | -0.02447315 | Tukey HSD | Table5 | 0.999915924 |
| 2 | 4 | 2.020833333 | 1.706896552 | 0.313936782 | 0.182467454 | 1.72050837 |  |  | 0.344752722 | Tukey HSD | Table5 | 0.423501744 |
| 2 | 5 | 2.020833333 | 2.11627907 | -0.095445736 | 0.196350922 | -0.486097725 |  |  | -0.09661456 | Tukey HSD | Table5 | 0.988578911 |
| 3 | 4 | 2.045454545 | 1.706896552 | 0.338557994 | 0.168303045 | 2.011597557 |  |  | 0.373743142 | Tukey HSD | Table5 | 0.263852822 |
| 3 | 5 | 2.045454545 | 2.11627907 | -0.070824524 | 0.183262729 | -0.386464421 |  |  | -0.073009227 | Tukey HSD | Table5 | 0.995247563 |
| 4 | 5 | 1.706896552 | 2.11627907 | -0.409382518 | 0.18818264 | -2.175453154 |  |  | -0.47496225 | Tukey HSD | Table5 | 0.192916768 |
| 1 | 2 | 3.440909091 | 3.613636364 | -0.172727273 | 0.220334123 | -0.783933375 |  |  | -0.292450221 | Tukey HSD | Table8 | 0.935061542 |
| 1 | 3 | 3.440909091 | 3.951101928 | -0.510192837 | 0.215091482 | -2.371980671 |  |  | -0.736161239 | Tukey HSD | Table8 | 0.126950615 |
| 1 | 4 | 3.440909091 | 4.01645768 | -0.575548589 | 0.217034534 | -2.651875623 |  |  | -0.982216097 | Tukey HSD | Table8 | 0.064735101 |
| 1 | 5 | 3.440909091 | 3.800211416 | -0.359302326 | 0.222531805 | -1.614611116 |  |  | -0.516331035 | Tukey HSD | Table8 | 0.489646572 |
| 2 | 3 | 3.613636364 | 3.951101928 | -0.337465565 | 0.120239794 | -2.806604656 |  |  | -0.522329499 | Tukey HSD | Table8 | 0.042971325 |
| 2 | 4 | 3.613636364 | 4.01645768 | -0.402821317 | 0.123682057 | -3.2569099 |  |  | -0.705156614 | Tukey HSD | Table8 | 0.011305422 |
| 2 | 5 | 3.613636364 | 3.800211416 | -0.186575053 | 0.133092699 | -1.401842881 |  |  | -0.293859555 | Tukey HSD | Table8 | 0.627136135 |
| 3 | 4 | 3.951101928 | 4.01645768 | -0.065355752 | 0.114080985 | -0.572889093 |  |  | -0.102218925 | Tukey HSD | Table8 | 0.978886821 |
| 3 | 5 | 3.951101928 | 3.800211416 | 0.150890512 | 0.124221119 | 1.214692906 |  |  | 0.2168439 | Tukey HSD | Table8 | 0.742865307 |
| 4 | 5 | 4.01645768 | 3.800211416 | 0.216246264 | 0.127555986 | 1.695304704 |  |  | 0.34454183 | Tukey HSD | Table8 | 0.438963886 |
| 1 | 2 | 3.493333333 | 3.394444444 | 0.098888889 | 0.29957095 | 0.33010173 |  |  | 0.128771388 | Tukey HSD | Table9 | 0.997421791 |
| 1 | 3 | 3.493333333 | 3.454545455 | 0.038787879 | 0.292442943 | 0.132634005 |  |  | 0.042331499 | Tukey HSD | Table9 | 0.999929837 |
| 1 | 4 | 3.493333333 | 3.002298851 | 0.491034483 | 0.295084758 | 1.664045564 |  |  | 0.584569052 | Tukey HSD | Table9 | 0.458402914 |
| 1 | 5 | 3.493333333 | 3.156589147 | 0.336744186 | 0.302558965 | 1.112986973 |  |  | 0.386568414 | Tukey HSD | Table9 | 0.799587402 |
| 2 | 3 | 3.394444444 | 3.454545455 | -0.06010101 | 0.163480575 | -0.367633953 |  |  | -0.068708814 | Tukey HSD | Table9 | 0.996082239 |
| 2 | 4 | 3.394444444 | 3.002298851 | 0.392145594 | 0.16816075 | 2.331968632 |  |  | 0.477302367 | Tukey HSD | Table9 | 0.138755389 |
| 2 | 5 | 3.394444444 | 3.156589147 | 0.237855297 | 0.180955658 | 1.314439677 |  |  | 0.284313649 | Tukey HSD | Table9 | 0.682538037 |
| 3 | 4 | 3.454545455 | 3.002298851 | 0.452246604 | 0.155106928 | 2.915708602 |  |  | 0.501693414 | Tukey HSD | Table9 | 0.031694912 |
| 3 | 5 | 3.454545455 | 3.156589147 | 0.297956307 | 0.168893669 | 1.76416504 |  |  | 0.322648755 | Tukey HSD | Table9 | 0.397223892 |
| 4 | 5 | 3.002298851 | 3.156589147 | -0.154290297 | 0.173427826 | -0.889651333 |  |  | -0.176376558 | Tukey HSD | Table9 | 0.900509939 |

A and B were in the TTM stage (1–5).
